# Removal of toxic hexavalent chromium ions from water using magnetic protic poly (ionic liquids) nanocomposites

**DOI:** 10.1038/s41598-026-51229-z

**Published:** 2026-06-11

**Authors:** Alia A. Melegy, E. G. Zaki, S. M. El-Saeed, Nermine E. Maysour, Yasser K. Abdel-Monem, Ayman M. Atta

**Affiliations:** 1 Petroleum Application Department, Egyption Petroleum Research Institute, Nasr City, 11727 Cairo Egypt; 2 Chemistry Department, Faculty of Science, Menoufia University, Al Minufiyah, Egypt

**Keywords:** Protic poly (ionic liquids), Magnetic nanocomposites, Ferrite, Heavy metals, Water pollutants, Adsorption, Chemistry, Environmental sciences, Materials science

## Abstract

Novel magnetic nanocomposites (MNCs) based on Fe_3_O_4_ and NiFe_2_O_4_ nanoparticles were synthesized at low temperature via an in-situ approach using linear and crosslinked quaternized protic poly (ionic liquid) (PIL) matrices. Linear PILs were prepared from quaternized triethanolammonium acrylate and quaternized triethanolammonium 2-acrylamido-2-methylpropane sulfonate to form LQAA, while crosslinked copolymerization with *N*,*N*′-methylenebisacrylamide yielded CQAA hydrogels. For comparison, acrylic acid-co-2-acrylamido-2-methylpropane sulfonic acid (CAA) hydrogels were also fabricated. The resulting MNCs were comprehensively characterized to elucidate their chemical structure, thermal stability, magnetic behavior, surface morphology, and crystallographic features. The nanocomposites exhibit superparamagnetic behavior, high structural stability, and uniform dispersion of ferrite nanoparticles within the ionic and hydrogel matrices. Their adsorption performance toward Cr ions removal from aqueous solutions was systematically investigated, including the effects of pH, contact time, and adsorbent dosage. The fate of Cr^6+^ in the aqueous solution, as well as the surface of the material, was determined; the results demonstrated not only the successful adsorption of Cr^6+^ from solution, but also confirmed the presence and in situ reduction of Cr^6+^ to Cr^3+^ on the surface. Kinetic and isotherm analyses reveal rapid adsorption governed by surface chemisorption and diffusion processes. Remarkably, the MNCs demonstrate exceptionally high equilibrium Cr ions adsorption capacities (800–950 mg g^−1^), exceeding those of most reported magnetic adsorbents. This superior performance arises from the synergistic combination of functionalized Fe_3_O_4_ and NiFe_2_O_4_ surfaces with highly charged PIL networks, which provide a high density of accessible binding sites. Competitive adsorption studies using simulated industrial wastewater containing Cr^6+^, Cu^2+^, and Ni^2+^ (up to 1000 mg L^−1^ each) further confirm the strong affinity, selectivity, and stability of the developed materials, particularly toward Cr ions. These findings highlight the potential of PIL-based magnetic nanocomposites as efficient, magnetically recoverable, and reusable adsorbents for advanced treatment of heavy metal contaminate.

**Supplementary Information:**

The online version contains supplementary material available at 10.1038/s41598-026-51229-z.

## Introduction

The rapid expansion of industrial activities such as electroplating, leather tanning, textile dyeing, mining, and pigment manufacturing has led to the continuous release of heavy metals into aquatic environments. Toxic metal ions, including Pb^2+^, Cd^2+^, Hg^2+^, Ni^2+^, Cu^2+^, and particularly hexavalent chromium ions (Cr^6+^, CrO_4_^2-^ and HCrO_4_^-^ pose severe environmental and health risks due to their persistence, bioaccumulation, and non-biodegradable nature^1,2^. Among them, Cr^6+^ is of special concern because of its high solubility, mobility, and carcinogenicity, whereas trivalent chromium (Cr^3+^ is comparatively less toxic and can be immobilized through precipitation or complexation^2,3^. Consequently, the effective removal of Cr^6+^ from wastewater remains a critical challenge in water treatment. Conventional remediation methods, including chemical precipitation, membrane filtration, ion exchange, and electrochemical processes, often suffer from limitations such as high cost, secondary sludge generation, membrane fouling, and poor selectivity at low metal concentrations^4^. The adsorption-based techniques have gained considerable attention owing to their operational simplicity, high efficiency, adaptability, and potential for adsorbent regeneration^5^. Accordingly, the development of advanced adsorbent materials with high capacity, rapid kinetics, selectivity, and easy recovery has become a major research focus. Hydrogel composites have emerged as promising adsorbents for heavy metal ions due to their high porosity, hydrophilicity, and abundance of functional groups (e.g., –COOH, –NH_2_, –OH, and –SO_3_H), which facilitate metal ion binding through chelation, ion exchange, and electrostatic interactions^6^. However, conventional hydrogels often exhibit poor mechanical strength and challenging separation from treated water. To address these drawbacks, magnetic nanocomposite (MNC) hydrogels have been developed by incorporating magnetic nanoparticles, such as Fe_3_O_4_ or ferrites, into polymeric hydrogel matrices^7^. The magnetic component enables rapid separation using an external magnetic field, eliminating the need for filtration or centrifugation^8^. Moreover, magnetic nanoparticles can enhance adsorption performance by increasing surface area and providing additional active sites, while the hydrogel matrix prevents aggregation and improves stability^9^. As a result, magnetic hydrogel nanocomposites have demonstrated high adsorption capacities and good reusability for heavy metals and oxyanions, including Cr^6+^^10^.

In parallel, ionic liquids (ILs) and polymeric ionic liquids (PILs) have attracted increasing interest for environmental remediation due to their strong solvation ability and affinity toward metal ions^11^. While the direct use of ILs is limited by high viscosity, cost, and potential leaching, PILs offer a robust alternative by combining the tunable chemistry of ILs with the mechanical stability of polymers^12,13^. PILs provide a high density of charged sites capable of interacting with metal ions via ion exchange, coordination, and electrostatic attraction, resulting in enhanced adsorption capacity and selectivity^14^. Integrating PILs with magnetic nanoparticles and hydrogel matrices yields PIL-based magnetic nanocomposite hydrogels that synergistically combine porosity, ionic functionality, and magnetic separability^15,16^. Such multifunctional systems are particularly effective for Cr^6+^ removal, where adsorption may be accompanied by partial reduction to Cr^3+^ and subsequent immobilization within the polymer matrix^17^. Several studies have reported superior Cr^6+^ adsorption performance for IL- or PIL-functionalized magnetic adsorbents, with high capacities over a wide pH range and good regeneration efficiency^18–24^. In-situ synthesis of magnetic nanocomposites, where nanoparticles nucleate and grow directly within a polymeric or ionic matrix, offers significant advantages over ex-situ approaches. This strategy enhances particle–matrix interactions, minimizes aggregation and leaching, and improves dispersion, stability, and functional performance^24,25^. Recent studies have demonstrated that protic and quaternized PIL networks can effectively cap and template the formation of ferrite nanoparticles, yielding well-dispersed, superparamagnetic composites without the need for high-temperature calcination^25,26^.

In our previous work linear and crosslinked acidic protic poly (ionic liquids), APILs, based on quaternization of triethanolamine with 2-acrylamido-2-methylpropane sulfonic acid (AMPS) co-acrylic acid (AA) to produce QAA copolymers, was prepared to act as capping agent to prepare photocatalysts based on zinc oxide, zinc ferrite and magnetite @zinc oxide nanocomposites^26^. The charged functional groups within hydrogel and PIL networks (e.g., sulfonate, carboxylate, and quaternary ammonium) act as coordination sites for metal precursors, promoting controlled nucleation and uniform nanoparticle distribution^24,26^. Against this background, the present work aims to design, synthesize, and evaluate novel magnetic nanocomposite adsorbents based on the same prepared APIL for efficient Cr^6+^ removal and recover from aqueous solutions by using an external magnet. Magnetite (Fe_3_O_4_) and nickel ferrite (NiFe_2_O_4_) nanoparticles were synthesized in-situ within polymeric matrices, including linear quaternized protic poly (ionic liquids) (LQAA), crosslinked quaternized acrylic-based hydrogels (CQAA), and crosslinked acrylic acid/sulfonated networks (CAA). The study focuses on elucidating structure–property relationships, particularly the influence of polymer ionic functionality, crosslink density, and nanoparticle distribution on swelling behavior, surface charge, magnetic separation, and adsorption performance.

This work introduces LQAA and CQAA as templet and synthesized Fe_3_O_4_ and NiFe_2_O_4_ nanoparticles with in-situ technique, enabling highly efficient Cr^6+^ removal through combined electrostatic adsorption and partial surface-mediated reduction. Unlike our previous work^24^, this study provides direct experimental evidence distinguishing Cr^6+^ adsorption from partial surface-mediated reduction through combined UV–Vis (DPC) and ICP-OES speciation analysis. Additionally, the comparison of Fe_3_O_4_^−^ and NiFe_2_O_4_-based hydrogels demonstrates the influence of metal substitution on surface charge, nanoparticle dispersion, and adsorption capacity, highlighting a rational design strategy for optimizing performance. This study represents a clear advancement over the previous works by addressing mechanistic and functional aspects of Cr^6+^ removal that were not explored before. By combining advanced material design, quantitative speciation, and mechanistic insight, this work not only achieves exceptionally high Cr^6+^ uptake but also provides a fundamental understanding of the pH-dependent adsorption–reduction interplay, setting it apart from prior publications and establishing it as a significant and novel contribution to the field of magnetic PIL-based water remediation Finally, the adsorption efficiency, kinetics, selectivity, and reusability of the developed magnetic nanocomposites for Cr^6+^ removal were systematically investigated to assess their potential for practical water treatment applications.

## Experimental

### Materials

All chemicals were of analytical grade and used as received without further purification. Acrylic acid (AA), 2-acrylamido-2-methylpropane sulfonic acid (AMPS), *N*,*N*′-methylenebisacrylamide (MBA), triethanolamine (TEA), and ammonium persulfate (APS) were purchased from Sigma-Aldrich Chemicals Co. Nickel chloride (NiCl_2_), anhydrous ferric chloride (FeCl_3_), sodium hydroxide (NaOH), potassium iodide (KI), and ammonium hydroxide solution (25 wt%) were used for the synthesis of magnetite and nickel ferrite nanocomposites. A stock solution of Cr^6+^ (1000 mg L^−1^) was prepared by dissolving analytical-grade K_2_Cr_2_O_7_ (2.828 g) in 1000 mL of deionized water. Working solutions of desired concentration (e.g., 10, 20, 30, 50, 60, 80, 100 and 200 mg L^−1^) were freshly prepared by dilution. Phosphate buffer solutions (PBS) were prepared using H_3_PO_4_/NaH_2_PO_4_ by titration of 0.1 N NaH_2_PO_4_ with 0.1 M HCl (pH 2–3) or 0.1 N NaOH (pH 7–12) until the desired pH was achieved. The pH was monitored using a calibrated pH meter. Deionized water was used throughout all experiments.

### Preparation technique

#### Preparation of linear quaternized protic poly (ionic liquid) (LQAA)

Linear quaternized protic poly (ionic liquid), LQAA, was synthesized via aqueous free-radical polymerization of in situ-generated quaternized acidic monomers under an oxygen-free atmosphere. Prior to use, all glassware was washed with dilute nitric acid, thoroughly rinsed with deionized (DI) water, and dried at 333 K. Monomers were handled under subdued light to minimize premature radical formation. In a representative synthesis, 2-acrylamido-2-methyl-1-propanesulfonic acid (AMPS, 0.10 mol, 20.72 g) was dissolved in 50 mL DI water in a three-necked round-bottom flask equipped with a mechanical stirrer, thermometer, and nitrogen inlet. Alternatively, acrylic acid (AA, 0.10 mol, 7.21 g) was used under identical conditions. The solution was cooled to 278 K in an ice bath, after which triethanolamine (TEA, 0.10 mol, 14.92 g) was added dropwise over 20 min with continuous stirring. The neutralization/quaternization mixture was maintained at 278 K for 24 h under gentle nitrogen flow to ensure complete proton transfer and formation of the quaternized ammonium monomer (QAMPS or QAA). Completion of monomer formation was confirmed by the appearance of a homogeneous, transparent amber solution and stabilization of pH (≈ 7–7.5). Ammonium persulfate (APS, 0.6 wt % relative to total monomer mass) was freshly dissolved in a minimal amount of DI water (2 mL) and introduced into the quaternized monomer solution under nitrogen. After 15 min degassing, polymerization was initiated at 278 K and the temperature was gradually increased to 333 K over 18 h using a programmable oil bath. This slow temperature ramp minimized localized radical concentration and prevented uncontrolled gelation, yielding a viscous, homogeneous polymer melt. For purification, the crude polymer was diluted with a minimal volume of DI water to reduce viscosity and filtered through a 0.45 µm hydrophilic membrane to remove dust and any coagulated fractions. The filtrate was transferred into dialysis tubing (molecular weight cut-off 3.5 kDa) and dialyzed against excess DI water for 72h at ambient temperature. The external water was replaced every 8 h until constant conductivity was reached, ensuring removal of residual TEA, APS fragments, and unreacted monomers. The dialyzed polymer solution was then concentrated under reduced pressure at 40–45 °C using a rotary evaporator and subsequently dried in a vacuum oven at 323 K to constant mass. A transparent amber viscous polymer (LQAA) was obtained in 98% yield. The material was stored in sealed amber glass vials under dry nitrogen to prevent moisture uptake.

#### Preparation and purification of crosslinked protic poly (ionic liquid) hydrogel (CPIL)

Crosslinked quaternized poly (ionic liquid) networks were prepared by free-radical copolymerization of the corresponding quaternized monomers in the presence of *N*,*N*′-methylenebisacrylamide (MBA) as a tetra-functional crosslinker. The quaternized monomers QAMPS and QAA were synthesized as described above and used as aqueous stock solutions. Typically, QAMPS solution (0.01 mol; 5 mL containing 3.57 g QAMPS) and QAA (0.01 mol; 2.22 g) were combined in a glass vial and stirred for 15 min to obtain a uniform precursor mixture. MBA (1 wt % relative to the combined mass of QAMPS and QAA) was dissolved separately in a small amount of DI water (1 mL) and added to the monomer mixture with stirring until fully dispersed. APS initiator (0.6 wt % relative to total monomer content) was then introduced as an aqueous solution. The reaction mixture was transferred into cylindrical glass molds (test tubes, inner diameter ≈ 10 mm). To remove dissolved oxygen, nitrogen was bubbled through the solution for 30 min using a fine needle while the molds were kept in an ice bath to suppress premature polymerization. After sealing, the molds were placed in a thermostated oven and heated gradually to 333 K, where they were maintained until solid hydrogel rods formed (12–18 h). The obtained rigid cylindrical gels were removed from the molds and post-cured at 378 K overnight to complete crosslinking and eliminate residual volatiles. The resulting quaternized crosslinked hydrogel was designated CQAA. For comparison, a non-quaternized network (CAA) was prepared using the same formulation and thermal schedule but employing unneutralized AMPS and AA (without TEA treatment). Purification of the hydrogels was carried out by exhaustive solvent extraction. The rods were cut into small discs (3–5 mm thickness) to increase diffusion area and immersed in excess DI water for 48 h with periodic water renewal (every 6 h) to remove soluble oligomers, residual initiator, and unreacted monomers. This was followed by extraction in absolute ethanol for 24 h to displace trapped water and extract remaining organic impurities. The cleaned gels were blotted, then dried in a vacuum oven at 323–333 K until constant weight was reached. Dried samples were stored in desiccators over silica gel prior to characterization.

#### Synthesis of magnetic nanocomposites

- Nanocomposites based on linear PIL (LQAA).

Ferric chloride (10 g, 0.06 mol) was dissolved in 75 mL of deoxygenated distilled water. KI solution (3.3 g, 0.02 mol in 15 mL water) was added dropwise under nitrogen, and the mixture was stirred for 1 h. The solid by-product was removed by filtration, and the clear filtrate was used as the iron precursor solution. LQAA (1.0 g, 10 wt% relative to iron content) was added under stirring, followed by dropwise addition of 100 mL aqueous ammonia (28 wt%). The mixture was stirred at 45 °C for 3 h under nitrogen and aged at 85 °C for 1 h to promote magnetite crystallization. The resulting Fe_3_O_4_·LQAA nanocomposites were magnetically separated, ultracentrifuged (8000 rpm, 20 min), washed repeatedly with ethanol/water (50:50 v/v) and absolute ethanol, and dried under vacuum at 40–50 °C for 24 h. Nickel ferrite nanocomposites (NiFe_2_O_4_·LQAA) were synthesized similarly using FeCl_3_ (0.04 mol) and Ni^2+^ salts (0.02 mol).

(b) Nanocomposites based on crosslinked PIL (CQAA).

Ferric chloride (10 g, 0.06 mol) and potassium iodide (0.02 mol) were reacted as described above to generate the iron precursor solution. After filtration, dried CQAA hydrogel pieces (0.50 g) were immersed in the filtrate and allowed to swell and complex iron ions for 24 h at room temperature with gentle stirring. The swollen gels were removed, briefly rinsed with ethanol to displace surface water, and air-dried to eliminate excess solvent while retaining the absorbed metal ions. The gels were then transferred to 100 mL of aqueous ammonia solution (28 wt%) and stirred for 3 h to induce in-situ precipitation of magnetite within the polymer network. The obtained magnetic hydrogels were repeatedly washed with ethanol/water (50:50 v/v) and then with absolute ethanol to remove unreacted ions, ammonia, and loosely bound particles. Drying was performed under reduced pressure at 40–50 °C for 24 h, yielding purified Fe_3_O_4_·CQAA nanocomposites. Nickel ferrite hydrogel nanocomposites (NiFe_2_O_4_·CQAA) were synthesized analogously by replacing part of Fe^3+^ (0.02 mol) with Ni^2+^ while maintaining a total metal ion content of 0.06 mol.

#### Characterization of synthesized ILs

The chemical structure of the prepared LQAA was identified from their ^1^H and ^13^C NMR spectra obtained on a JEOL-ECS 400 MHz spectrometer at ambient temperature using residual solvent signals as internal standards (DMSO-d6: ^1^H, 2.50 ppm; ^13^C, 39.52 ppm). Attenuated total reflectance–Fourier-transform spectroscopy (ATR–FTIR) analyses were carried out over the wavenumber range of 4000–400 cm^−1^ using a Thermo Scientific Nicolet iS10 analyzer. The morphology and crystal size distribution of the MNCs were examined using complementary transmission electron microscopy techniques, including a JEOL 3010 operating at 297 kV, a Philips CM20 operating at 200 kV, and a JEOL 2100 TEM operating at 200 kV. Particle size measurements were carried out using two-dimensional projections of the grains obtained from bright-field TEM images, which were analyzed using ImageJ software. Size distributions were determined by fitting ellipses to the crystal outlines, and the particle diameter was estimated from the average of the ellipse axes. Approximately 150 particles were measured for each sample. Bright-field TEM images were used to investigate the morphology of the synthesized products. High-resolution TEM images were recorded to determine lattice parameters and examine the crystal structure. In addition, selected-area electron diffraction patterns were collected to identify individual crystals and measure their lattice parameters. The scanning electron microscopy (SEM-JEOL JXA-840A) instrument at 20 kV was used to determine the surface morphology, composition and the particle dispersion or agglomeration of the samples. Elemental and chemical analysis was quantified using the electron dispersive X-ray (EDX). The crystal size, and diffraction pattern of nanocomposites were performed using a Bruker D2 X-ray powder diffractometer (30 kV, 10 mA) using Cu anode (k = 0.15406 nm) at 25 °C. N_2_ adsorption–desorption isotherms were recorded at 77 K with an automated sorption analyzer (Micromeritics ASAP 2000) after degassing the samples at 150 °C overnight under nitrogen flow. The pore size distribution was determined using the Barrett–Joyner–Halenda (BJH) method, while the specific surface area and pore volume were calculated by the Brunauer–Emmett–Teller (BET) technique. Thermal stability was assessed through thermogravimetric and differential thermogravimetric analysis (TGA-DTG; TGA-50 SHIMADZU) under a nitrogen atmosphere. Briefly, about 10 mg of the sample was heated to 800 °C at a heating rate of 10 °C/min. Vabritaing Sample Magnotometer (VSM) analyses were performed using a LakeShore VSM system to determine the magnetic properties of MNCs. Inductively coupled plasma optical emission spectroscopy (ICP-OES, Agilent 5800, USA) was used to examine the total heavy metal concentrations in aqueous solutions such as Cr^6+^, Cr^3+^, Cu^2+^, Ni^2+^ and catalyst leaching in water during photodegradation. UV–visible spectrophotometer (Shimadzu UV-1208 model) was used to determine the concentration of Cr^6+^. Zeta potential (mV) of MNCs at different pH of aqueous solutions were determined using (model NanoPlus-3, Nano Plus series from Particulate Systems). The leaching of magnetite from polymer composites at different pH was detected using the same technique.

### Adsorption of Cr ions and heavy metals on MNCs

#### Determination of Cr^6+^ and Cr^3+^ concentrations

To distinguish between Cr^6+^ adsorption and possible reduction to Cr^3+^, a combined UV–Vis spectrophotometric and ICP-OES analytical approach was employed. A stock solution of Cr^6+^ (1000 mg L^−1^) was prepared by dissolving analytical-grade K_2_Cr_2_O₇ (2.828 g) in 1000 mL of deionized water. Working solutions of desired concentration (e.g., 200 mg L^−1^) were freshly prepared by dilution. The solution pH was adjusted to 2.0 ± 0.1 using 0.1 M H_2_SO_4_ to ensure Cr^6+^predominately existed as HCrO_4_^−^ species. In a typical experiment, 20 mg of the MNCs was added to 50 mL of Cr^6+^ solution (200 mg L^−1^) in a sealed conical flask. The suspension was shaken at 200 rpm at 25 °C for 120 min (previously determined equilibrium time). The MNCs adsorbent was separated magnetically. The supernatant was filtered through a 0.22 µm membrane filter. The filtrate was immediately analyzed. All experiments were performed in triplicate. Concentration Cr^6+^ concentration was determined using the 1,5-diphenylcarbazide (DPC) colorimetric method. In this respect 1 mL of 0.25% (w/v) DPC solution (prepared in acetone) was added to each10 mL of filtrate and 1 mL of 1 M H_2_SO_4_ was added to maintain acidic conditions. After 10 min reaction time, absorbance was measured at 540 nm using a UV–Vis spectrophotometer. Calibration curves were prepared using standard Cr^6+^ solutions (0–5 mg L^−1^). Cr^3+^ does not react with DPC under these conditions and therefore does not interfere with Cr^6+^ quantification. Total chromium (Cr_total_) concentration was determined by ICP-OES. Samples were acidified with 2% HNO_3_ prior to measurement. ICP-OES quantifies total elemental chromium regardless of oxidation state. The concentration of Cr^3+^ formed during adsorption was calculated indirectly using the relation: [Cr^3+^] = [Cr_total_]–[Cr^6+^].

#### Adsorption and desorption experiments

The adsorption parameters of MNCs for MB metal ions Cr^6+^, Cu^2+^ and Ni^2+^ were investigated in the batch experiments at 25 °C. The dry MNCs samples (0.01–0.05 g) were immersed in 100 mL of Cr ions(its concentration is 1000 mg L^−1^) at optimum pH (5.8) and stirred at room temperature for 24 h. The effect of the initial Cr ions concentration on the adsorption capacity was investigated by changing the concentrations from 100 to 2000 mg L^−1^ in different buffer solutions. ICP-OES was used to examine the heavy metal contents in aqueous solutions Different concentrations of Cr ions from 100 to 1000 ppm dissolved in 50 mL of PBS and stirred with 0.02 g of the MNCs into a 100 mL conical flask at 25 °C. The remained Cr ions concentration was determined using ICP-OES after filtration and magnetic separation using external magnet. The amount of Cr ions adsorption at equilibrium q_e_ (mg/g) and percent extraction (%E) was calculated from the following equations:

1 $${\mathrm{q}}_{{\mathrm{e}}} = [\left( {{\mathrm{C}}_{{\mathrm{o}}} - {\mathrm{C}}_{{\mathrm{e}}} } \right) \times {\mathrm{V}}/\left( {\mathrm{m}} \right)]$$

2 $$\% {\mathrm{E}} = [\left( {{\mathrm{C}}_{{\mathrm{o}}} - {\mathrm{C}}_{{\mathrm{e}}} } \right) \times {1}00/\left( {{\mathrm{C}}_{{\mathrm{o}}} } \right)]$$

where Cₒ and C_e_ (mg/L) are the liquid phase concentrations of Cr ions at initial and equilibrium, respectively, V (L) the volume of the solution and m (g) is the mass of adsorbent used. All the experiments were repeated three times and average values were reported. The standard deviation was found to be from ± 0.5 to ± 2.12%; values of correlation coefficient were in the range 0.98–0.99.

At the end of the adsorption period, the Cr ions adsorbed nanocomposite adsorbent was isolated from the mixture by decantation and then was treated with five portions of 20 mL 2 M HNO_3_. The suspensions were shaken on a rotary shaker at 150 rpm for 24 h. Then, part of the regenerated sorbent is treated with 0.1 M NaOH to restore the negative charge on the nanocomposite. Then they were placed in distilled water for a week to reach swelling equilibrium and were subjected to a second-time adsorption. In the same way, consecutive adsorption–desorption cycles were repeated four times by using the same nanocomposite hydrogel so that the reusable adsorption could be determined. Reloading efficiency was calculated using the following equation:

$${\mathrm{Reloadingefficiency}} = {\text{amountofCradsorbedafterreuse/amountofCradsorbed before reuse}}.$$

The same technique for desorption of Cr ions was applied to MNCs instead NaOH solution (10 mL, 0.1–0.3 M) were used to desorb the metal ions. Adsorption–desorption cycles were repeated three times using the same MNCs.

#### Multi-component heavy metal adsorption

Adsorption of heavy metal ions from synthetic wastewater was carried out in a batch system. The concentration of metal ions in synthetic wastewater is 1000 mg L^−1^ for each of Ni^2+^, Cu^2+^ and Cr^6+^. A stock solution of Cr^6+^ (1000 mg L^−1^) was prepared by dissolving analytical-grade K_2_Cr_2_O_7_ (2.828 g) in 1000 mL of deionized water. Synthetic Cu^2+^ and Ni^2+^ solutions were prepared from copper sulfate pentahydrate (CuSO_4_·5H_2_O; 3.93 g in 1 L) and nickel nitrate hexahydrate (Ni(NO_3_)_2_·6H_2_O; 4.96 g in 1 L), respectively (purity ≥ 99%). In order to adjust salinity, 700 ppm of NaCl was added to the synthetic wastewater. A dry MNC sample (0.1 g) was transferred into 100 mL of synthetic wastewater for 1000 ppm of Ni^2+^, Cu^2+^ and Cr^6+^, stirred and incubated at room temperature (pH 5.8). After adsorption, the concentration of the metal ions in the remaining solution was determined by ICP-OES as described above after removal the MNCs by using an external magnet.

## Results and discussion

Conventional magnetic nanoparticle synthesis methods, such as co-precipitation and sol–gel routes, often suffer from weak polymer–nanoparticle interactions, leading to aggregation and diminished functional performance. Ex-situ incorporation of preformed nanoparticles into polymer matrices typically results in poor interfacial adhesion and limited stability. In contrast, in-situ synthesis within ionic or hydrogel networks enables controlled nucleation, strong particle–matrix interactions, and improved dispersion, which collectively enhance mechanical and functional properties. This advantage has been widely reported, with in-situ precipitation in hydrogel matrices producing significantly more homogeneous and well-dispersed nanoparticles than ex-situ approaches^25,26^. Embedding magnetic nanocomposites (MNCs) within LPILs and crosslinked hydrogels is particularly attractive for applications such as catalysis and environmental remediation, where high surface area and facile magnetic separation are essential. Previous studies demonstrated that protic PIL-based systems allow in-situ formation of magnetically immobilized nanoparticles that exhibit excellent catalytic degradation of organic dyes, such as methylene blue, under UV irradiation, highlighting their potential for wastewater treatment applications^25,26^. In earlier work, superparamagnetic magnetite nanoparticles were synthesized via a modified coprecipitation route involving FeCl_3_ and KI, followed by alkaline treatment with ammonia. This method produced high yields of pure magnetite without formation of secondary iron oxides over a temperature range of 45–65 °C, depending on the capping agent used^24,26^. The formation of magnetite proceeds according to the following reactions:

3 $${\mathrm{3Fe}}^{{{3} + }} + {\text{ I}}^{ - } \to {\text{ 2Fe}}^{{{3} + }} + {\text{ Fe}}^{{{2} + }} + { 1}/{\mathrm{2I}}_{{2}}$$

4 $${\mathrm{2Fe}}^{{{3} + }} + {\text{ Fe}}^{{{2} + }} + {\text{ 8OH}}^{ - } \to {\text{ Fe}}_{{3}} {\mathrm{O}}_{{4}} + {\text{ 4H}}_{{2}} {\mathrm{O}}$$

**Scheme 1:**
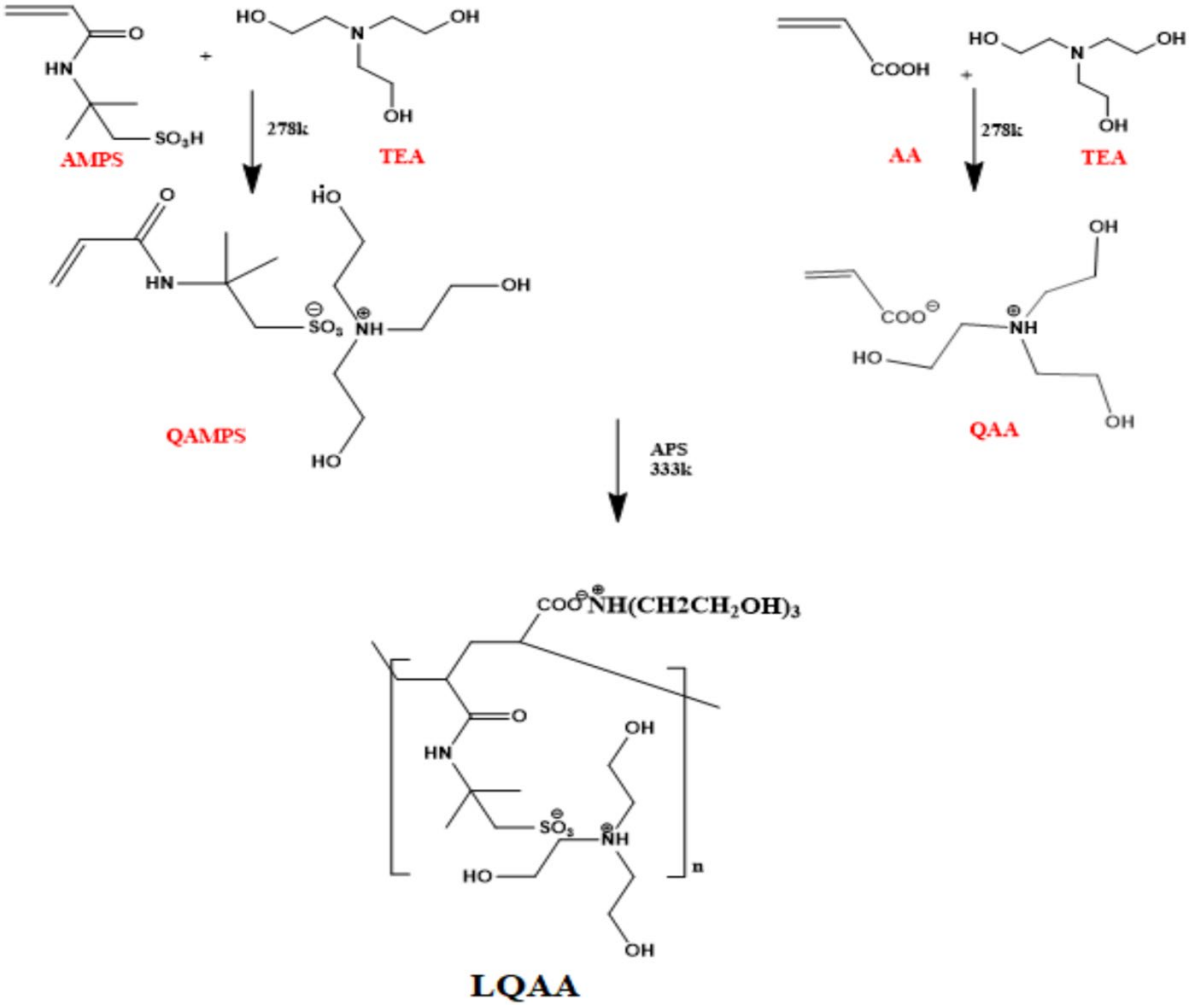
Synthesis of LQAA polymer.

The same procedure was used to prepare Ni.Fe_2_O_4_ by mixing 2 mol of FeCl_3_ with one mol of NiCl_2_ instead 3 mol of FeCl_3_ as reported in the experimental part. Acidic protic poly (ionic liquids), APILs, based on quaternization of triethanolamine with 2-acrylamido-2-methylpropane sulfonic acid (AMPS) co-acrylic acid (AA), was prepared according to Scheme 1 to apply as capping agent for magnetite or nickel ferrite nanomaterials. APILs were easily prepared with moderate viscosity and used as good capping agent to produce superior magnetic materials due to presence either ionic sulfonic, and carboxylic or nonionic hydroxyl and amide groups that bound metal ions to prevent the formation of other iron oxides such as α-Fe_2_O_3_ (hematite, weakly ferromagnetic or antiferromagnetic), γ-Fe_2_O_3_ (maghemite, ferrimagnetic), FeO (wüstite, antiferromagnetic), ε-Fe_2_O_3_ and β-Fe_2_O^24,26^**.** APILs act as stabilizing agents and templates during the co-precipitation process. They control the nucleation and growth of NiFe_2_O_4_ nanoparticles by providing a confined ionic environment. APILs also influence the surface chemistry of the nanoparticles, introducing functional groups that can enhance their performance in various applications.

**Scheme 2.**
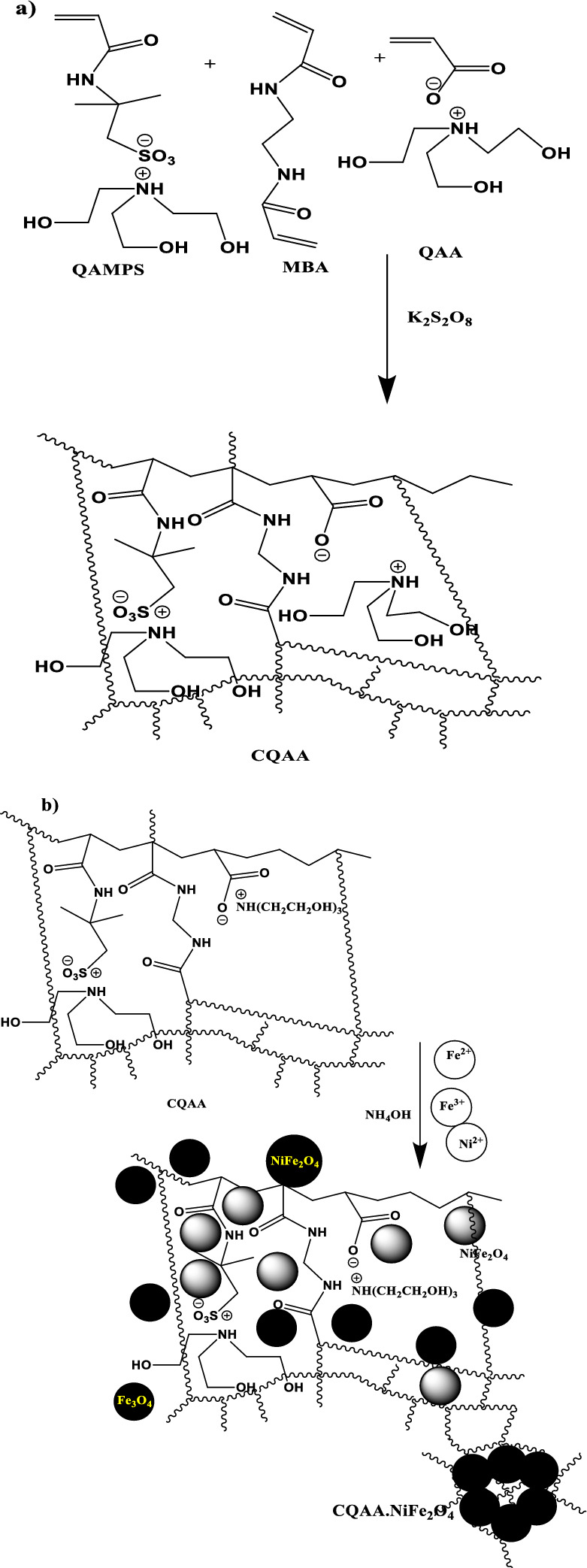
The crosslinking copolymerization of (**a**) CQAA and (**b**) CQAA.Ni.Fe_2_O_4_ magnetic nanocomposite.

The crosslinking of QAMPS and QAA in the presence of MBA yielded well-defined three-dimensional CQAA hydrogel networks (Scheme 2a). The incorporation of permanent quaternary ammonium groups significantly increased the charge density within the polymer matrix, providing abundant binding sites for Fe^2+^, Fe^3+^, and Ni^2+^ ions. Compared with non-quaternized analogues, CQAA hydrogels exhibited stronger metal ion affinity due to the combined effects of sulfonate, acrylate, amide, and triethanolammonium groups, which promote chelation, ion exchange, and hydrogen bonding. Subsequent alkaline treatment of the metal-loaded hydrogels induced in-situ formation of magnetite or nickel ferrite nanoparticles within the polymer network (Scheme 2b). This in-situ strategy enables precise control over metal content, particle distribution, and ferrite composition, offering a versatile platform for tuning magnetic and adsorption properties. Consequently, the prepared MNCs are promising candidates for water purification applications, particularly for the efficient removal of heavy metal ions and other charged contaminants. Moreover, APIL-assisted synthesis is expected to yield superparamagnetic nanoparticles with controlled particle size, narrow size distribution, and improved crystallinity compared with conventional co-precipitation methods, as discussed in the following sections.

It is expected that the presence of APILs based on CQAA and LQAA produce valuable superparamagnetic nanoparticles having controlled morphologies, particle and crystal sizes based on APIL type as discussed in the next section. This method offers enhanced control over particle size, morphology, and crystallinity. It is expected that, NiFe_2_O_4_ synthesized using PILs often exhibits smaller particle sizes, narrower size distributions, and better crystallinity compared to materials prepared by conventional co-precipitation methods. The APILs works synergistically to control the nucleation and growth processes, resulting in superior material properties.

### Characterization of MNCs

**Fig. 1:**
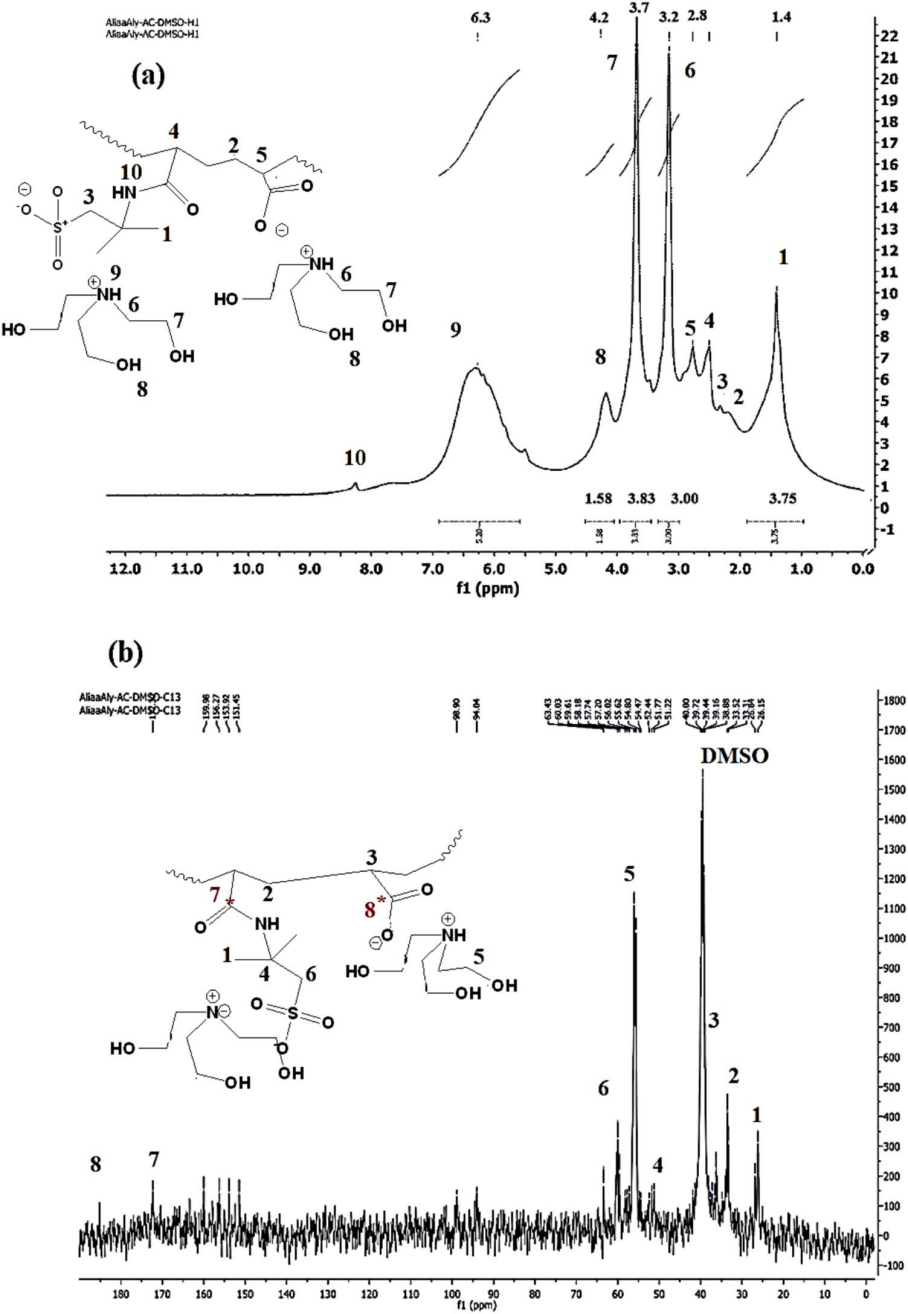
NMR spectra of LQAA (QAMPS/QAa)**a**) ^1^HNMR and (**b**) ^13^CNMR using deuterated DMSO as solvent.

The ^1^H NMR spectrum of the LQAA (recorded in DMSO-d6) represented in Fig. 1a confirms successful copolymer formation. The chemical structure of LQAA correlated with their peaks was illustrated in Fig. 1a,b. A broad resonance at ~ 6.0–6.5 ppm is assigned to the amide NH proton of the AMPS unit, broadened due to hydrogen bonding and ionic interactions. Signals at ~ 3.7–4.2 ppm and ~ 3.0–3.3 ppm correspond to the O–CH_2_ and N–CH_2_ protons of the triethanolammonium cation, respectively. Broad multiplets in the range of ~ 1.5–2.5 ppm are attributed to the polymer backbone (–CH_2_– and –CH–) protons. The methyl groups of the AMPS C(CH_3_)_2_ moiety appear at ~ 1.2–1.4 ppm. Notably, the absence of proton signals (≈4.5–6.5 ppm) confirms complete consumption of the monomer double bonds. Moreover, the absence of carboxylic protons of acrylic moieties at chemical shift from 10 to 12 ppm confirm its quaternization with triethaolamine. Collectively, the spectral features verify incorporation of both monomeric units and preservation of the ionic structure within the copolymeric framework.

The ^13^CNMR spectrum of the LQAA (recorded in DMSO-d6) summarized in Fig. 1b confirms successful copolymer formation. Two distinct carbonyl signals at ~ 175–178 ppm and ~ 170–174 ppm are assigned to the amide (AMPS) and carboxylate (acrylate) groups, respectively. The absence of vinyl carbon resonances (≈125–135 ppm) indicates complete consumption of the monomer double bonds. Broad resonances between 35–45 ppm correspond to the polymer backbone (–CH– and –CH_2_–). Signals at ~ 58–62 ppm and ~ 52–56 ppm are attributed to O–CH_2_ and N–CH_2_ carbons of the triethanolammonium cation. The methyl carbons of the AMPS 2-methylpropane moiety appear at ~ 22–25 ppm. In the ^13^C NMR spectrum (Fig. 1b), a signal at ~ 170–174 ppm corresponds to the carboxylate (COO^−^) carbon, signals at ~ 52–56 ppm and ~ 58–62 ppm are assigned to N–CH_2_ and O–CH_2_ carbons of the protonated triethanolammonium cation. In the ^1^H NMR spectrum (Fig. 1a), resonances at ~ 3.0–3.3 ppm and ~ 3.7–4.0 ppm correspond to N–CH_2_ and O–CH_2_ protons, respectively. The absence of a –COOH proton signal supports the presence of the deprotonated carboxylate form. Together, these signals confirm formation of the acrylate triethanolammonium ionic structure within the copolymer.

**Fig. 2:**
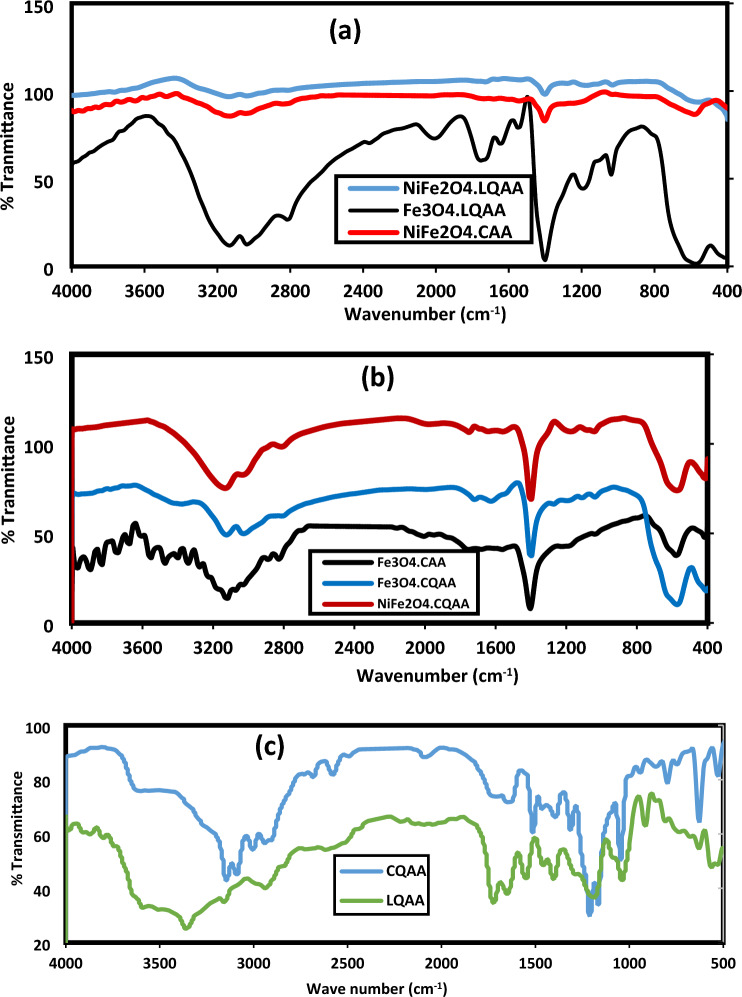
FTIR (**a**) Fe_3_O_4_, (**b**) and NiFe_2_O_4_ embedded into LQAA, CQAA or CAA networks and (**c**) pure CQAA and LQAA spectra.

Fourier-transform infrared spectroscopy (FTIR) was employed to investigate surface functionalization, chemical bonding, and polymer–nanoparticle interactions in Fe_3_O_4_ and NiFe_2_O_4_ nanocomposites prepared with LQAA, CQAA, and CAA matrices. The FTIR spectra of Fe_3_O_4_^−^ and NiFe_2_O_4_^−^ based nanocomposites are shown in Fig. 2a,b, while spectra of pristine LQAA, and CQAA hydrogels are represented in Fig. 2c. The frequencies of the absorption bands of MNCs capped with LQAA, CQAA and CAA hydrogels were summarized in Table 1. FTIR analysis provides direct evidence of the spinel ferrite structure and confirms the successful capping of nanoparticles by PIL networks. Characteristic metal–oxygen (M–O) vibrations of spinel ferrites appear in the 400–600 cm^−1^ region. For NiFe_2_O_4_, strong absorption bands between 400 and 600 cm^−1^ are attributed to Fe^3+^–O and Ni^2+^–O stretching vibrations at tetrahedral and octahedral sites^27^. The intense bands below 610 cm^−1^ confirm the preservation of the spinel ferrite core after polymer capping. The gradual shift of the Fe–O band from 580 cm^−1^ (Fe_3_O_4_) toward ~ 560 cm^−1^ with increasing Ni content, together with the increasing intensity of the Ni–O band near 450 cm^−1^, confirms the formation of spinel NiFe_2_O_4_. Broad absorption bands in the 3400–3200 cm^−1^ range are assigned to O–H and N–H stretching vibrations arising from adsorbed water, surface hydroxyl groups, and amide functionalities of the polymer matrices. These bands also reflect strong hydrogen bonding between triethanolammonium cations and ferrite surfaces. Aliphatic C–H stretching bands at 2920–2850 cm^−1^ confirm the presence of polymer backbones and successful copolymerization. The amide I band (C=O stretching) at 1620–1650 cm^−1^ further confirms incorporation of acrylamide units on the nanoparticle surface. The sulfonate (SO_3_^−^) groups exhibit characteristic bands in the 1040–1200 cm^−1^ (symmetric) and 1350–1450 cm^−1^ (asymmetric) regions. A prominent band at 1401 cm^−1^ observed in all nanocomposites (Fig. 2a,b) is attributed to the partially ionized S=O stretching vibrations of the anchored bidentate sulfonate groups interacting with surface Fe^3+^ or Ni^2+^ ions^28,29^**.** This peak serves as a clear signature of successful surface functionalization by sulfonated ionic liquids, confirming strong polymer–nanoparticle interactions.

**Table 1:** FTIR peaks assignments of MNCs and their PILs.

Assignments	Vibration type	Frequencies/cm^−1^			Frequencies/cm^−1^			Frequencies/cm^−1^		
		LQAA	Fe_3_O_4_	NiFe_2_O_4_	CQAA	Fe_3_O_4_	NiFe_2_O_4_	CAA	Fe_3_O_4_	NiFe_2_O_4_
M–OH (water)		–	3680	3700	–	3500	–	3655	3670	3729
–OH	Ѵ_O–H_	3650	3300–3133	3137	3600	3123	3131	3500–3100	3470	3131
	δ_O–H_	915	–	–	942					
–NH	Ѵ_+N–H_	3358	3100–3010	3038	3150			–		
	δ_+N–H_	1640	1644	1640	1649			–		
–CH	Ѵ_C–H_	2934	2900	2850	2941	2875	2850	2946	2827	2954
	δ_C–H_	1403	1350	1375	1394			1375		
HN-C=O	Ѵ_CON–H_(I)	1645	1644	1640	1645	1626	1650	1665	1650	
	Ѵ_CON–H_(II)	1552	1544	1540	1512	1550	1559	1513	1563	1539
HO-C=O	Ѵ_COO_^−^	1723	1700	1727	1705	1700	1750	1724		
	δ_COO_^−^	1186	1193	1174	1168			1168		
C–N+	Ѵ_C–N_^+^	1457	–	–	1470			1309		
M-S=O		–	1401	1402	1394	1401	1401	–	1401	1401
SO_3_^−^	ν_as_ _SO3_^−^	1220	1193	1174	1210		1174	1220		
	ν_s__SO3_^−^	1039	1038	1030	1047		1043	1042		
Fe–O			580	558	–	580	571	–	576	579
Ni–O			–	420	–		420	–		430

**Fig. 3:**
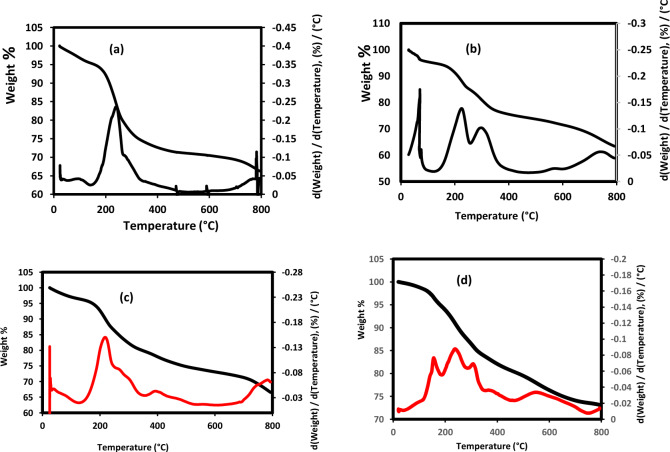
TGA-DTG thermograms of (**a**) Fe_3_O_4_.LQAA, (**b**) NiFe_2_O_4_.LQAA, (**c**) Fe_3_O_4_.CQAA, and (**d**) NiFe_2_O_4_.CQAA nanocomposites.

The thermal stability and organic content of Fe_3_O_4_ and NiFe_2_O_4_ nanocomposites were examined by TGA–DTG analysis. The corresponding thermograms for LQAA- and CQAA-capped samples are shown in Fig. 3a–d, while those of pristine polymers are provided in supplementary file Fig. S1. DTG profiles enable identification of distinct decomposition stages associated with surface-bound species and polymer matrices. All samples exhibit multiple weight-loss steps. The initial loss below 150 °C is attributed to desorption of physically adsorbed water and residual solvents. The second stage (200–400 °C) corresponds to decomposition of ionic polymer components and organic residues, accompanied by dehydration of surface –OH groups within the spinel structure. Above 400–650 °C, the materials become thermally stable, indicating formation of Fe_3_O_4_ and NiFe_2_O_4_ spinel phases. Ferrites synthesized in CQAA networks show enhanced thermal stability compared to LQAA-capped counterparts. Fe_3_O_4_–CQAA samples contain 5 wt.% adsorbed water, whereas NiFe_2_O_4_–CQAA exhibits negligible surface water. The initial degradation temperature of NiFe_2_O_4_–CQAA (275 °C) is markedly higher than that of NiFe_2_O_4_–LQAA (227 °C), confirming the stabilizing effect of crosslinked polyionic networks through strong polymer–nanoparticle interactions. Residual masses at 650 °C (Rs, wt.%) reflect the inorganic ferrite content. Fe_3_O_4_–LQAA shows residual masses of 68.03 and 63.22 wt.% (Fig. 3a,b), while Fe_3_O_4_–CQAA and NiFe_2_O_4_–CQAA exhibit residual masses of 67.3 and 73.6 wt. % (Fig. 3c,d), that corrected to be 50.33 and 57.3 wt.% after subtracting the residual polymer contribution (Fig. S1b). The higher residual content of NiFe_2_O_4_ agrees with the stronger Fe–O and Ni–O bands observed in FTIR spectra (Fig. 2a,b). NiFe_2_O_4_ samples display negligible mass loss above 600 °C, confirming complete decomposition by 560 °C and formation of pure, thermally stable NiFe_2_O_4_ without further oxidation^30^.

**Fig. 4:**
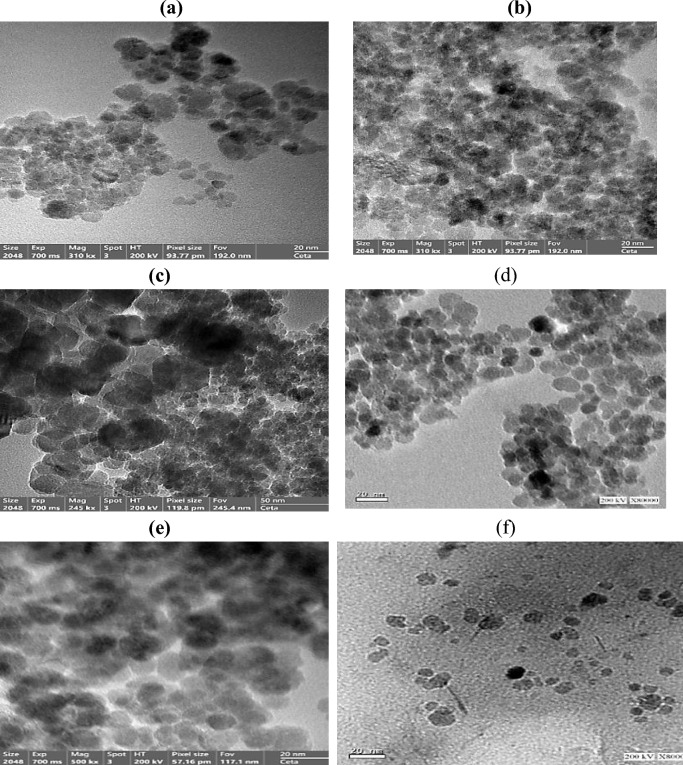
TEM micrographs of (**a**) Fe_3_O_4_.LQAA, (**b**) NiFe_2_O_4_.LQAA, (**c**) Fe_3_O_4_.CQAA, (**d**) NiFe_2_O_4_.CQAA (**e**) Fe_3_O_4_.CAA, (**f**) NiFe_2_O_4_.CAA.

**Fig. 5:**
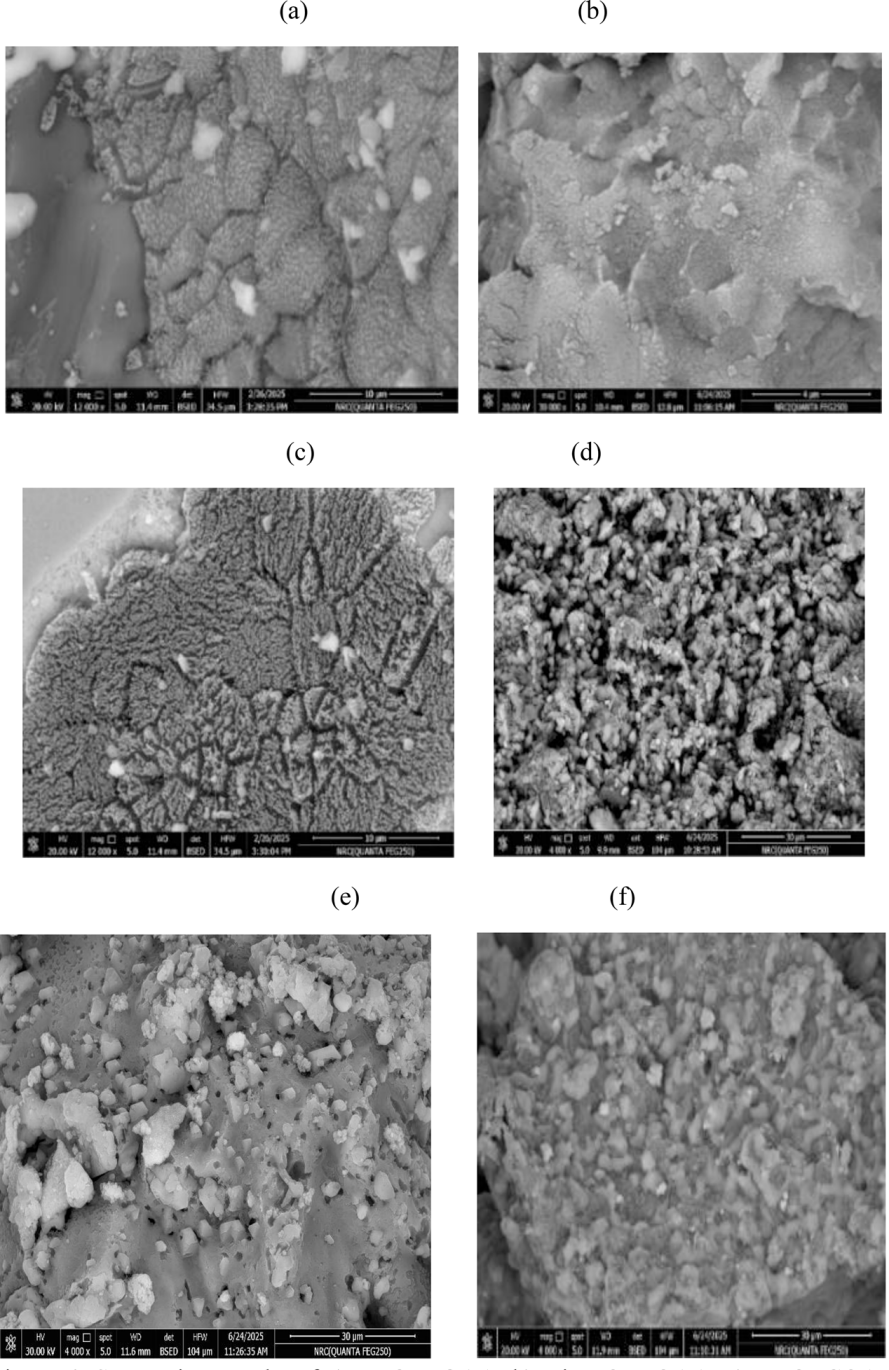
SEM micrographs of (**a**) Fe_3_O_4_.LQAA, (**b**) NiFe_2_O_4_.LQAA, (**c**) Fe_3_O_4_.CQAA,(Fe3O4.CPIL) [26], (**d**) NiFe_2_O_4_.CQAA (**e**) Fe_3_O_4_.CAA, (**f**) NiFe_2_O_4_.CAA.

The morphology, particle size, and dispersion state of the synthesized magnetic nanocomposites (MNCs) were examined by TEM and SEM, as shown in Figs. 4a–f and 5a–f. These complementary techniques provide detailed insight into the nanoscale characteristics of the ferrite nanoparticles and their microscale organization within different polymer matrices.

TEM images of Fe_3_O_4_.LQAA and NiFe_2_O_4_.LQAA (Fig. 4a,b) reveal quasi-spherical nanoparticles with relatively narrow size distributions and clear contrast between the inorganic cores and the surrounding polymer phase. The average particle size lies within the nanometric range (6–12 nm), consistent with magnetite and nickel ferrite nanoparticles synthesized via wet-chemical routes and stabilized by polymeric capping agents^31–33^. Partial aggregation into small clusters is observed in the LQAA systems, which can be attributed to magnetic dipole–dipole interactions combined with the limited steric hindrance provided by flexible linear polymer chains. Similar clustered morphologies have been reported for Fe_3_O_4_ nanoparticles coated with linear polyelectrolytes or ionic polymers^34,35^. The crosslinked systems (Fe_3_O_4_.CQAA and NiFe_2_O_4_.CQAA; Fig. 4c,d) exhibit a more homogeneous nanoparticle distribution with significantly reduced aggregation. The polymer network effectively immobilizes individual particles, indicating strong interfacial interactions between quaternized ammonium groups, sulfonate moieties, and ferrite surfaces. Crosslinking restricts particle mobility during growth and drying, thereby limiting secondary aggregation and promoting uniform dispersion^36^. The CAA-based nanocomposites (Fe_3_O_4_.CAA and NiFe_2_O_4_.CAA; Fig. 4e,f) show the highest degree of dispersion, with nanoparticles embedded within a continuous hydrogel matrix and only minor clustering. The high density of –COOH and –SO_3_H groups in the acrylic acid/AMPS network provides multiple coordination and hydrogen-bonding sites, enhancing electrostatic stabilization and suppressing nanoparticle coalescence. Comparable behavior has been reported for ferrite nanoparticles incorporated into poly (acrylic acid) and sulfonated hydrogel systems. Across all matrices, NiFe_2_O_4_ nanoparticles are slightly smaller and more uniformly distributed than Fe_3_O_4_, reflecting differences in nucleation and growth kinetics of nickel ferrite spinel structures under similar synthesis conditions.

SEM analysis further elucidates the surface morphology of the MNCs (Fig. 5a–f). Fe_3_O_4_-based composites (Fe_3_O_4_.LQAA and Fe_3_O_4_.CQAA; Fig. 5a,c) display irregular, rough surfaces with micron-scale undulations and pronounced granular features. These textures suggest partial nanoparticle aggregation within the polymer matrix, likely driven by strong polar interactions between magnetite surfaces and quaternized ionic groups. Such surface heterogeneity is consistent with previous reports on magnetite–polymer composites, where limited steric stabilization leads to rough, granular morphologies^37,38^. In contrast, NiFe_2_O_4_-based samples (NiFe_2_O_4_.LQAA and NiFe_2_O_4_.CQAA; Fig. 5b,d) exhibit smoother and more homogeneous surfaces composed of densely packed, fine features. This morphology reflects a finer dispersion of spinel ferrite domains and reduced local agglomeration, likely arising from improved compatibility between NiFe_2_O_4_ surfaces and the polar ionic polymer backbone. Similar observations have been reported for NiFe_2_O_4_ nanoparticles embedded in hydrophilic polymer matrices^39^. Morphological differences are further pronounced when comparing linear LQAA matrices with crosslinked hydrogels (CQAA and CAA). The crosslinked systems consistently show more interconnected and textured surfaces with reduced particle agglomeration. Fe_3_O_4_.CAA and NiFe_2_O_4_.CAA (Fig. 5e,f) exhibit rugged, sponge-like morphologies, characteristic of three-dimensional polymer networks that enhance nanoparticle anchoring while suppressing large cluster formation. This behavior is consistent with previous studies on hydrogel-based magnetic nanocomposites, where crosslinking enhances dispersion, surface roughness, and network stability^40^. Overall, SEM results confirm that both ferrite composition and polymer architecture play critical roles in determining surface morphology. Crosslinked hydrogels (CQAA and CAA) promote improved nanoparticle distribution and higher surface complexity, which are advantageous for applications requiring high surface area and active interaction sites. Combined TEM and SEM analyses demonstrate that polymer architecture dominates nanoparticle dispersion behavior: linear LQAA matrices permit limited aggregation, whereas CQAA and especially CAA networks effectively immobilize and separate nanoparticles. Ferrite composition further influences morphology, with NiFe_2_O_4_ consistently forming smaller and more uniformly dispersed particles than Fe_3_O_4_, in agreement with previous reports on spinel ferrite/polymer nanocomposites^21,23,26^. These findings confirm the successful formation of well-dispersed Fe_3_O_4_ and NiFe_2_O_4_ nanoparticles within tailored ionic and hydrogel matrices and highlight the effectiveness of quaternized PILs and acrylic acid/AMPS hydrogels as stabilizing platforms for advanced magnetic nanocomposites.

**Fig. 6:**
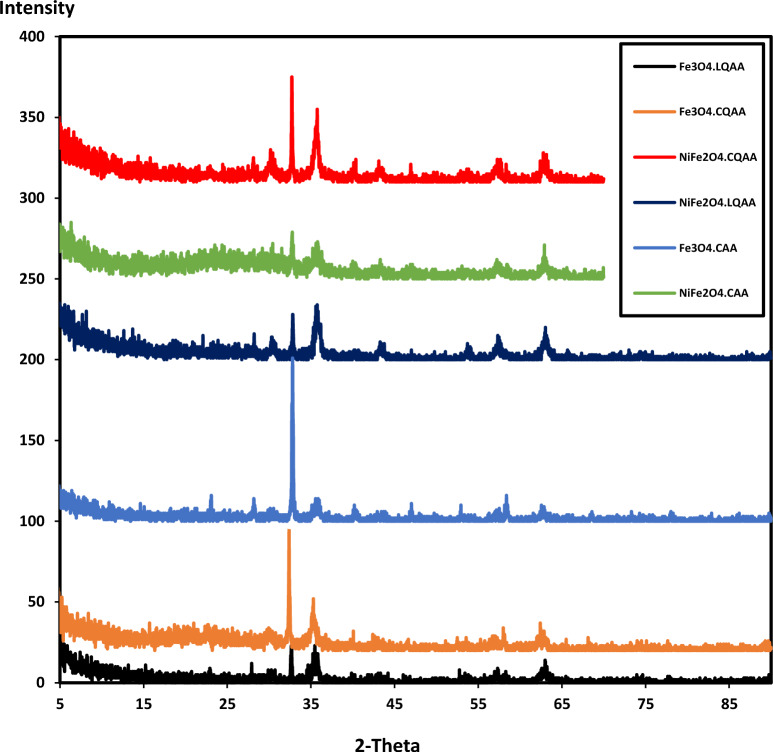
XRD diffractograms of the prepared Fe_3_O_4_ and NiFe_2_O_4_ nanocomposites.

X-ray powder diffraction (XRD) was employed to examine the crystal structure, phase purity, and crystallite characteristics of Fe_3_O_4_ and NiFe_2_O_4_ nanoparticles synthesized within LPIL and hydrogel matrices. Figure 6 shows the XRD patterns of the magnetic nanocomposites, while the diffractograms of pristine CQAA and CAA polymers are presented in Fig. S2.

All Fe_3_O_4_-based samples (Fe_3_O_4_.LQAA, Fe_3_O_4_.CQAA, and Fe_3_O_4_.CAA) exhibit diffraction peaks at 2θ ≈ 30.2°, 35.5°, 43.2°, 53.5°, 57.1°, and 62.7°, corresponding to the (220), (311), (400), (422), (511), and (440) planes of cubic inverse-spinel magnetite (JCPDS No. 19-0629). These reflections confirm the formation of phase-pure Fe_3_O_4_, consistent with previous reports on polymer-supported magnetite nanoparticles^31–33^. Likewise, NiFe_2_O_4_ nanocomposites (NiFe_2_O_4_.LQAA, NiFe_2_O_4_.CQAA, and NiFe_2_O_4_.CAA) display characteristic peaks at 2θ ≈ 30.3°, 35.7°, 37.3°, 43.3°, 53.7°, 57.3°, and 62.9°, indexed to the (220), (311), (222), (400), (422), (511), and (440) planes of cubic spinel NiFe_2_O_4_ (JCPDS No. 10-0325)^39^. The absence of secondary phases in all samples confirms high phase purity. In all nanocomposites, the sharp ferrite reflections are superimposed on a broad diffuse halo centered at 2θ ≈ 18–25°, arising from the amorphous polymer matrices (LQAA, CQAA, and CAA). This coexistence of crystalline ferrite and amorphous polymer is typical of magnetic polymer nanocomposites and has been widely reported^41,42^. Compared to LQAA samples, CQAA and CAA composites exhibit slightly reduced peak intensities and increased peak broadening, indicative of smaller crystallite sizes and stronger polymer–nanoparticle interactions within crosslinked networks. The higher amorphous content in these systems further enhances background scattering, reducing the relative intensity of ferrite peaks. NiFe_2_O_4_-based composites generally show broader diffraction peaks than their Fe_3_O_4_ counterparts, suggesting smaller average crystallite sizes and higher lattice strain. This behavior is consistent with the faster nucleation and finer crystallite formation typically observed for nickel ferrite under comparable synthesis conditions^40^. The relatively higher intensity of the (311) reflection in NiFe_2_O_4_ further reflects its well-ordered spinel structure, in agreement with earlier studies on polymer-supported NiFe_2_O_4_ nanocomposites. These XRD observations correlate well with TEM and SEM results (Figs. 4 and 5). The peak broadening observed for CQAA and CAA systems corresponds to the smaller, more uniformly dispersed nanoparticles detected by TEM and the smoother, more homogeneous surfaces observed by SEM. In contrast, the sharper reflections of LQAA samples reflect partial aggregation and larger effective crystallite domains, consistent with microscopic observations.

**Fig. 7:**
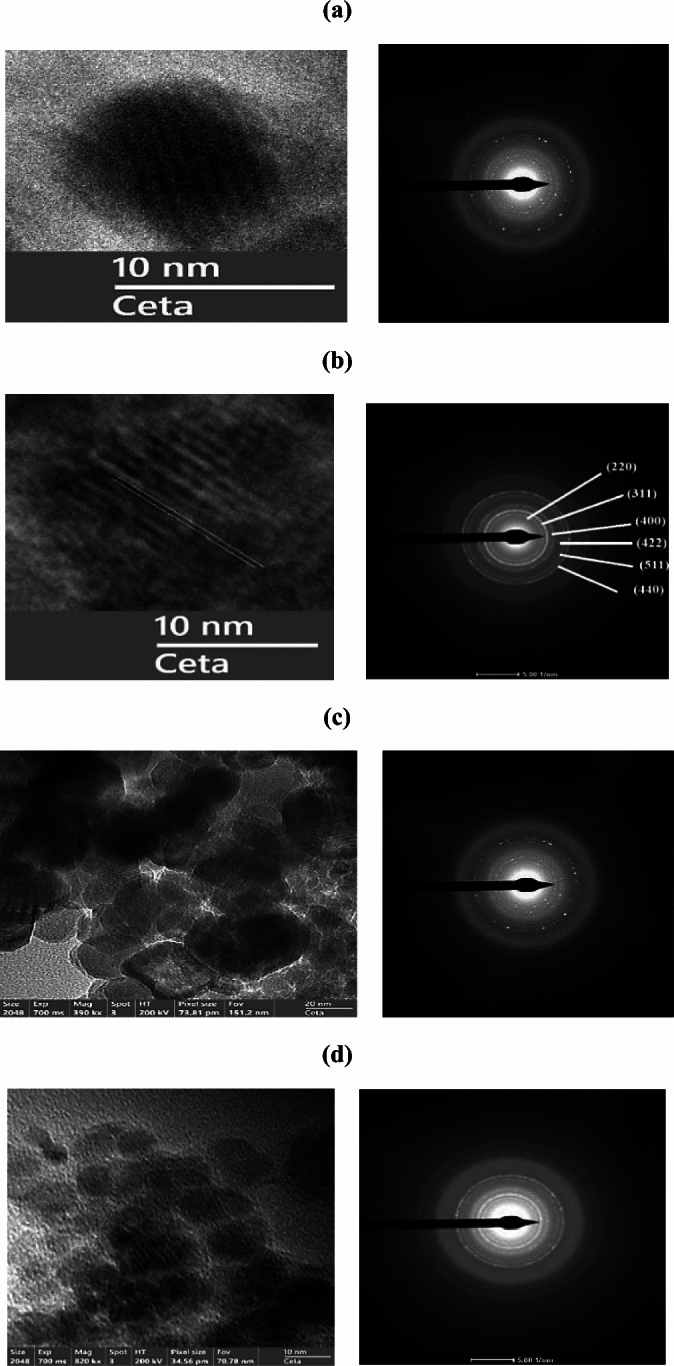
HRTEM and SAED analyses of (**a**) Fe_3_O_4_.LQAA, (**b**) NiFe_2_O_4_.LQAA, (**c**) Fe_3_O_4_.CQAA, and (**d**) Fe_3_O_4_.CAA MNcs.

Further structural confirmation was obtained from HRTEM and selected area electron diffraction (SAED), summarized in Fig. 7. SAED patterns consist of concentric diffraction rings, indicating polycrystalline nanoparticles with random orientations. For Fe_3_O_4_ composites, the rings correspond to the (220), (311), (400), (422), (511), and (440) planes of cubic magnetite, while NiFe_2_O_4_ samples show rings indexed to the (220), (311), (222), (400), (422), (511), and (440) planes of cubic nickel ferrite, in excellent agreement with XRD results^40–42^. Notably, SAED rings in CQAA and CAA systems appear broader and more diffuse than those of LQAA samples, reflecting reduced coherent scattering domains and increased nanoscale disorder due to polymer confinement. HRTEM images reveal clear lattice fringes in all samples, confirming high crystallinity of individual nanoparticles. Fe_3_O_4_ nanocomposites show an interplanar spacing of ~ 0.253 nm, corresponding to the (311) plane of magnetite, while NiFe_2_O_4_ samples exhibit spacings of 0.249–0.252 nm, consistent with the (311) plane of cubic nickel ferrite^40–43^. The preservation of lattice fringes demonstrates that neither ionic polymer capping nor crosslinked hydrogel matrices disrupt the intrinsic spinel structure of the ferrite cores. Overall, XRD, SAED, and HRTEM analyses provide consistent evidence that Fe_3_O_4_ and NiFe_2_O_4_ nanoparticles retain their cubic spinel structures within LQAA, CQAA, and CAA matrices. Crosslinked polymer networks effectively reduce crystallite size and increase structural disorder without inducing phase transformation, while NiFe_2_O_4_ consistently forms smaller crystalline domains than Fe_3_O_4_, in line with known spinel ferrite growth kinetics^21,23,43^. Together with SEM and TEM results, these findings demonstrate that CQAA and CAA matrices offer superior control over nanoscale structure and dispersion compared to linear PIL systems, making them promising platforms for advanced magnetic nanocomposite applications.

The preservation of clear lattice fringes demonstrates that neither the ionic polymer capping (LQAA) nor the crosslinked hydrogel matrices (CQAA and CAA) disrupt the intrinsic crystal structure of the ferrite cores. Furthermore, CQAA and CAA systems show slightly smaller crystalline domains in HRTEM images compared with LQAA samples, corroborating both the XRD and SAED results. This reduction in crystalline domain size is attributed to the restricted diffusion of metal ions and the steric confinement imposed by the crosslinked polymer network during nanoparticle nucleation and growth^21,23^. The structural information obtained from XRD, SAED, and HRTEM is highly consistent. XRD confirms long-range crystallographic order and phase purity of Fe_3_O_4_ and NiFe_2_O_4_. SAED verifies the polycrystalline nature and spinel structure at the local scale. HRTEM directly reveals lattice fringes and interplanar spacings matching the dominant XRD reflections. Together, these results demonstrate that both magnetite and nickel ferrite nanoparticles retain their cubic spinel structures after incorporation into LPIL, CQAA, and CAA matrices. Crosslinked polymer networks reduce crystallite size and increase structural disorder without inducing phase transformation. NiFe_2_O_4_ nanoparticles consistently show slightly smaller crystalline domains than Fe_3_O_4_, in agreement with previous reports on spinel ferrite growth kinetics^43^. The combined XRD–SAED–HRTEM analysis confirms the successful fabrication of highly crystalline Fe_3_O_4_ and NiFe_2_O_4_ nanoparticles uniformly stabilized within quaternized PIL and hydrogel networks. The polymer matrices do not alter the intrinsic crystal structure but significantly influence crystallite size and structural ordering. These structural characteristics, together with the previously discussed SEM and TEM morphologies, demonstrate that CQAA and CAA matrices provide superior nanoscale control compared to linear LPIL systems, making them promising platforms for advanced magnetic nanocomposite applications such as adsorption, catalysis, sensing, and biomedical delivery.

### Magnetic properties, porosity and surface charges of MNCs

**Fig. 8:**
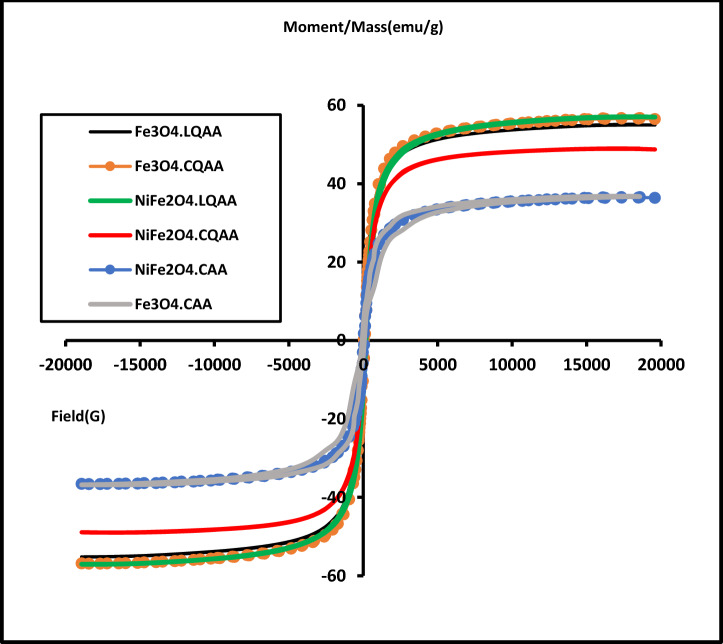
VSM hysteresis loops of the prepared MNCs based on Fe_3_O_4_ and NiFe_2_O_4_ at room temperature.

Vibrating sample magnetometry (VSM) was employed to evaluate the magnetic properties of Fe_3_O_4_ and NiFe_2_O_4_ nanoparticles capped with PILs, including saturation magnetization (M_s_), remanent magnetization (M_r_), and coercivity (Hc). The saturation magnetization (M_s_) represents the maximum magnetization attained under an applied magnetic field, while the remanent magnetization (M_r_) is the residual magnetization after field removal and is closely related to magnetic anisotropy and interparticle interactions. The coercivity (Hc) reflects the resistance of the material to demagnetization. Room-temperature VSM hysteresis loops of Fe_3_O_4_ and NiFe_2_O_4_ capped with LQAA, CQAA, and CAA are shown in Fig. 8, and the corresponding magnetic parameters are summarized in Table 2. All samples exhibit S-shaped (sigmoidal) magnetization curves with negligible hysteresis, indicating typical superparamagnetic behavior. The very low M_r_ and Hc values (close to zero) confirm that all nanocomposites behave as soft magnetic materials. Both Fe_3_O_4_ and NiFe_2_O_4_ capped with LQAA display nearly identical magnetic behavior, characterized by low coercivity and high saturation magnetization (Fig. 8, Table 2). The extremely small Hc values are attributed to the nanoscale dimensions of the ferrite particles, where domain wall motion is suppressed, as also evidenced by TEM observations (Figs. 4 and 7). Notably, NiFe_2_O_4_ capped with LQAA exhibits an M_s_ value higher than or comparable to many previously reported NiFe_2_O_4_ nanostructures and even bulk nickel ferrite (56 emu g^−1^), including single-crystalline nano-octahedra (55.1 emu g^−1^)^44^, hollow nanospheres (49.5–56.5 emu g^−1^)^29^, nanorods (40.9–44.2 emu g^−1^)^30^, hollow nanoleaves and nanoparticles (44.7–46.5 emu g^−1^)^45^, and nanowires/nanochains (43 emu g^−1^)^46^. The M_s_ value of NiFe_2_O_4_ capped with LQAA is lower than that of NiFe_2_O_4_ nanospheres synthesized by solvothermal methods with larger particle sizes (140–165 nm), but higher than values reported for spherical nanoparticles with sizes below 20 nm^47^. These enhancements are attributed to the controlled growth environment provided by PILs, which optimizes the cation distribution (Ni^2+^/Fe^3+^) within the spinel lattice. In addition, the presence of potassium iodide (KI) may further enhance M_s_ by influencing the oxidation states and site occupancy of metal ions during synthesis. Fe_3_O_4_ and NiFe_2_O_4_ nanoparticles capped with CQAA also exhibit superparamagnetic behavior with very low M_r_ and Hc values. However, a clear reduction in M_s_ is observed for CQAA-capped nanocomposites compared with their LQAA-capped counterparts. This decrease is attributed to the higher polymer content and thicker capping layer in CQAA systems, as confirmed by TGA-DTG analysis (Fig. 3). The magnetic properties of the polymer-supported magnetic nanocomposites show a direct correlation with the ferrite content in the composite matrix. As the ferrite wt.% decreases (e.g., from 68 wt.% to 40 wt.% for Fe_3_O_4_-based systems), a systematic reduction in saturation magnetization (Ms) is observed, which is expected due to magnetic dilution by the non-magnetic polymer phase and partial surface spin disorder at the polymer–nanoparticle interface. This behavior follows the rule of mixtures, where Ms of the composite scales proportionally with the magnetic phase fraction, provided no significant phase transformation occurs. The concomitant decrease in remanent magnetization (Mr) and coercivity (Hc) at lower ferrite loading suggests reduced interparticle dipolar interactions and improved magnetic isolation by the polymer coating. Similar trends have been widely reported for ferrite–polymer nanocomposites, where decreasing magnetic content and enhanced surface capping lead to suppressed magnetic anisotropy and reduced magnetostatic coupling while preserving soft ferromagnetic or near-superparamagnetic behavior at room temperature^26–28,44–47^. The extensive polymer coverage can form a magnetically “dead layer” at the particle surface, where spin disorder or canting reduces the effective magnetic moment. Furthermore, NiFe_2_O_4_ capped with CQAA shows lower M_s_ values than magnetite nanoparticles capped with CAA, highlighting the stronger shielding effect of the crosslinked CQAA network. In contrast, LQAA provides better surface passivation while minimizing spin disorder and aggregation, resulting in higher M_s_ values^48–50^. HRTEM images (Fig. 7) reveal well-defined lattice fringes for all samples, indicating good crystallinity, which correlates with enhanced magnetic performance. Overall, PILs play a crucial role in reducing coercivity by promoting the formation of smaller, more uniform nanoparticles with fewer structural defects. They also influence M_r_ by controlling interparticle spacing and suppressing agglomeration, leading to nearly zero remanence in well-dispersed systems. The combined effects of PIL architecture and KI addition provide an effective strategy for tailoring the magnetic properties of NiFe_2_O_4_ and Fe_3_O_4_ nanocomposites, making them promising candidates for applications in magnetic storage, catalysis, and environmental water pollutant adsorption and separation.

**Table 2:** Magnetic characteristics of the prepared Fe_3_O_4_ and NiFe_2_O_4_ at room temperature obtained from their VSM hysteresis loops and correlated with their TGA-DTG data.

Magnetic properties			Ferrite type		Capping PILs
Hc G	Mr emu g^−1^	Ms emu g^−1^	Content (wt.%)	Type	
7.28 ± 0.03	0.68 ± 0.005	55.18 ± 0.06	68.03 ± 3.5	Fe_3_O_4_	LQAA
14.18 ± 0.02	1.07 ± 0.003	57.07 ± 0.04	63.22 ± 1.2	Ni.Fe_2_O_4_	
12.04 ± 0.01	0.93 ± 0.002	56.81 ± 0.02	50.33 ± 1.8	Fe_3_O_4_	CQAA
12.57 ± 0.05	0.87 ± 0.003	48.97 ± 0.01	57.30 ± 2.4	Ni.Fe_2_O_4_	
5.87 ± 0.02	0.32 ± 0.003	36.81 ± 0.03	40.41 ± 1.4	Fe_3_O_4_	CAA
5.97 ± 0.03	0.53 ± 0.006	36.95 ± 0.05	45.32 ± 2.8	Ni.Fe_2_O_4_	

**Fig. 9:**
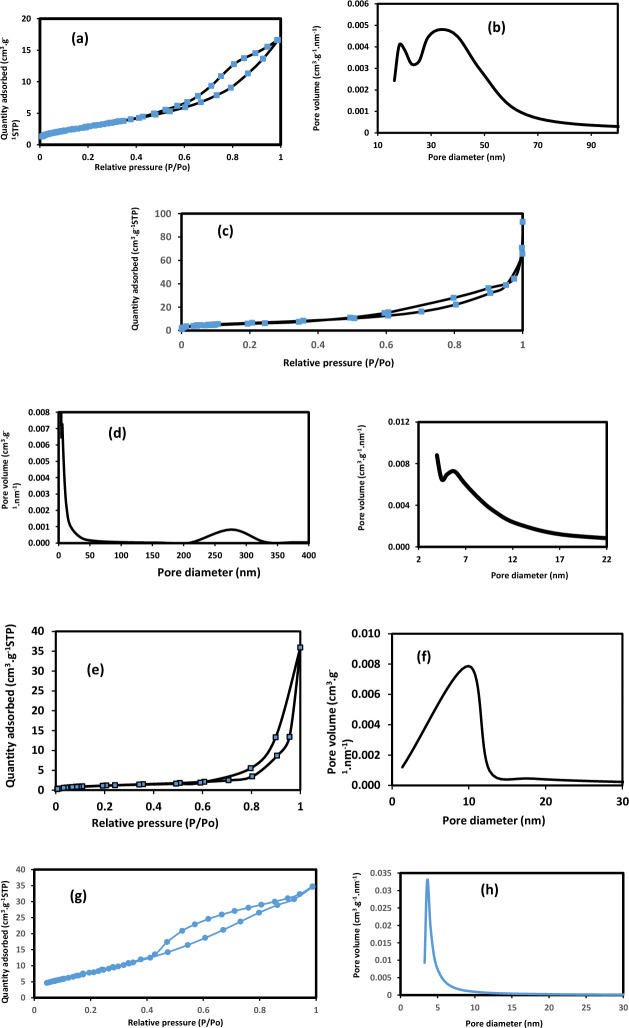
BET curves (**a**) N_2_ adsorption–desorption isotherm, (**b**) pore size distribution hysteresis loop of Fe_3_O_4_.LQAA, (**c**) and (**c**) N_2_ adsorption–desorption isotherm, (**d**) pore size distribution hysteresis loop of NiFe_2_O_4_.LQAA, (**e**) N_2_ adsorption–desorption isotherm, (**f**) pore size distribution hysteresis loop of Fe_3_O_4_.CQAA, (**g**) N_2_ adsorption–desorption isotherm, and (**h**) pore size distribution hysteresis loop of NiFe_2_O_4_.CQAA at room temperature.

BET analysis is a critical tool for evaluating the porosity and surface area of materials, since a larger surface area provides more active sites, which enhances their absorption capacities. Furthermore, an optimized pore structure facilitates the diffusion of adsorptive molecules toward active sites, thereby improving overall adsorption efficiency. The textural parameters of all synthesized samples are summarized in Table 3. BET analysis of the nanocomposites were selected as representative and summarized in Fig. 9a–h.

**Table 3:** BET data of the prepared nanocomposites.

BET data			PIL composites
Average pore diameter (nm)	Total pore volume (cm^3^ g^−1^)	Surface area (m^2^ g^−1^)	
33.786	0.202	105.1	Fe_3_O_4_.LQAA
12.36	0.0932	30.169	NiFe_2_O_4_.LQAA
21.61	0.1275	53.88	Fe_3_O_4_.CQAA
3.607	0.056	42.846	NiFe_2_O_4_.CQAA

The N_2_ adsorption–desorption isotherms (Fig. 9a–f) exhibit a type IV profile with a pronounced hysteresis loop at intermediate–high relative pressures, confirming the mesoporous nature of the material. The BJH pore size distribution of Fe_3_O_4_.LQAA (Fig. 9b) shows a dominant pore diameter centered at ~ 30 nm with a minor population around 15–20 nm and a tail extending toward larger pores. These results indicate a hierarchical mesoporous structure composed of primary mesopores and interparticle voids. The N_2_ adsorption–desorption isotherm of NiFe_2_O_4_.LQAA (Fig. 9 c) confirms the mesoporous nature of the material. The moderate adsorption at low relative pressure (P/P₀ < 0.3) suggests minimal microporosity, while the gradual increase in the intermediate region (0.4–0.0.8 corresponds to multilayer adsorption on mesopore walls. The sharp rise near P/P₀ ≈ 0.9–1.0 indicates capillary condensation in larger mesopores and interparticle voids. The presence of a hysteresis loop further supports mesoporosity arising from aggregated nanoparticles. This interpretation is strongly supported by the pore size distribution curves (Fig. 9d). The dominant pore population lies within the mesoporous range (≈3–10 nm), as shown by the high pore volume in this region. The declining tail toward larger pore diameters indicates broader mesopores and possibly interparticle macroporous voids. The absence of a significant peak below 2 nm confirms negligible microporosity, consistent with the limited adsorption at low P/P₀ in the isotherm. The N_2_ adsorption–desorption isotherm of Fe_3_O_4_.CQAA (Fig. 9e) exhibits a typical type IV isotherm, characteristic of mesoporous materials. The gradual adsorption at low relative pressure (P/P₀ < 0.3) indicates limited microporosity and monolayer–multilayer adsorption on the external surface. The pronounced increase in adsorbed volume at high relative pressure (P/P₀ ≈ 0.8–1.0) suggests capillary condensation within mesopores. The presence of a hysteresis loop confirms a mesoporous structure, likely associated with slit-shaped or interparticle pores formed by nanoparticle aggregation. Its pore size distribution curve (Fig. 9f) shows a dominant narrow peak centered around approximately 7–10 nm, confirming that the material is predominantly mesoporous. The small tail extending toward larger pore diameters indicates minor contribution from wider mesopores or interparticle voids. The absence of significant pore volume below 2 nm further supports negligible microporosity. Overall, the BET data indicate a mesoporous material with relatively uniform pore size distribution and pore structure governed mainly by interparticle mesoporosity rather than intrinsic micropores. The nitrogen adsorption–desorption isotherm of the NiFe_2_O_4_.CQAA (Fig. 9g) exhibits a type IV curve with a distinct hysteresis loop, confirming the mesoporous nature of the nanocomposite. The hysteresis resembles an H3-type loop, indicating slit-shaped pores formed by the aggregation of nanoparticles. The pore size distribution of the NiFe_2_O_4_.CQAA (Fig. 9h) shows a sharp maximum centered at approximately 3–4 nm, demonstrating a narrow mesopore distribution with negligible macroporosity.

BET analysis data (Table 3) of the MNCs capped with LQAA or CQAA demonstrates pronounced agreement in textural properties compared with their SEM images (Fig. 5a–d) and confirming the strong dependence of textural properties on morphology and polymer type. The Fe_3_O_4_.LQAA sample exhibits a highly porous, branched, and loosely packed structure, which is consistent with its highest surface area (105.1 m^2^ g^−1^) and large pore volume (0.202 cm^3^ g^−1^), indicating the formation of accessible mesoporous networks. NiFe_2_O_4_.LQAA shows a relatively denser and less developed porous texture in SEM, correlating with its significantly lower surface area (30.169 m^2^ g^−1^) and pore volume (0.0932 cm^3^ g^−1^). For the CQAA-based composites, Fe_3_O_4_.CQAA displays moderately aggregated particles with visible interparticle voids, matching its intermediate BET values (53.88 m^2^ g^−1^ and 0.1275 cm^3^ g^−1^). Meanwhile, NiFe_2_O_4_.CQAA presents a more compact and tightly agglomerated morphology with limited visible porosity, which explains its reduced pore volume (0.056 cm^3^ g^−1^) and smaller average pore diameter (3.607 nm). The SEM observations confirm that increased aggregation and structural compactness lead to decreased surface area and pore accessibility, whereas more open and hierarchical structures promote enhanced textural properties. The BET data (Table 3) elucidates that Fe_3_O_4_ capped with either LQAA or CQAA have larger surface area than NiFe_2_O_4_ MNCs which enhanced with functionalization of MNCs with LQAA. The lowering of surface area is due to the immobilization of CQAA or LQAA in the pores of MNCs. The good functionalization of nanocomposites with the ILs decreased the tangling and folding of the nanoparticles, and as a result, enhanced their accessibility for nitrogen adsorption^51^.

The surface charges of the prepared MNCs were measured at different pH solutions having 0.01M ionic strength and summarized in Table 4. The surface charges ζ-potential data (Table 4) demonstrate a clear pH-dependent surface charge behavior governed by the protonation/deprotonation of surface hydroxyl groups on Fe_3_O_4_/NiFe_2_O_4_ and the ionizable groups of the polymer shells. For LQAA- and CQAA-capped systems containing triethanolammonium moieties, highly positive ζ-potentials are observed at pH 2 (e.g., + 60.2 mV for LQAA–Fe_3_O_4_), reflecting protonation of surface ≡Fe–OH groups (≡Fe–OH_2+_) and ammonium functionalities. With increasing pH, progressive deprotonation of surface hydroxyls and carboxyl/sulfonate groups shifts the ζ-potential toward near-neutral values at pH 6 and negative values at pH 9, consistent with the amphoteric nature of spinel ferrites and the acid–base equilibria of –COOH/–SO_3_H groups. This trend agrees with the reported isoelectric points (IEP) of Fe_3_O_4_ (pH 6–7) and NiFe_2_O_4_ (pH 6–8), above which surfaces become negatively charged due to ≡M–O^−^ formation^52^. While, CAA-capped composites exhibit negative ζ-potentials across the entire pH range, even at pH 2, indicating dominance of sulfonate/carboxylate groups and effective surface coverage by the crosslinked acidic network, which suppresses the intrinsic IEP of the ferrite cores. The increasingly negative values at higher pH (down to − 42 mV at pH 9) reflect extensive deprotonation of –SO_3_H and –COOH groups, enhancing electrostatic stabilization. Such polymer-induced IEP shifts and charge reversal effects are well documented for carboxylate/sulfonate-functionalized iron oxides^53,54^.

**Table 4:** Zeta potential of the prepared Fe_3_O_4_ and NiFe_2_O_4_ MNCs at different pH aqueous solutions.

Zeta potentials (mV) after Cr ions adsorption	Zeta potentials (mV) at different pH aqueous solutions			Ferrite type	Capping PILs
pH = 5.8	pH = 9	pH = 6	pH = 2		
− 31.21 ± 2.13	− 22.89 ± 1.53	8.93 ± 0.5	60.22 ± 1.66	Fe_3_O_4_	LQAA
− 9.79 ± 1.29	− 10.50 ± 0.82	0.36 ± 0.03	44.83 ± 1.74	Ni.Fe_2_O_4_	
− 20.73 ± 2.89	− 29.20 ± 1.91	1.04 ± 0.02	35.22 ± 1.52	Fe_3_O_4_	CQAA
− 30.9 ± 2.35	− 12.12 ± 1.15	1.80 ± 0.03	17.2 ± 1.41	Ni.Fe_2_O_4_	
− 13.79 ± 1.74	− 42.35 ± 3.72	− 30.55 ± 1.23	− 20.05 ± 2.13	Fe_3_O_4_	CAA
− 10.65 ± 1.25	− 41.82 ± 2.83	− 27.01 ± 1.06	− 14.36 ± 1.35	Ni.Fe_2_O_4_	

### Application of MNCs as adsorbent for Cr^6+^

The efficient removal of hexavalent chromium (Cr^6+^) and other heavy metals from wastewater remains a major challenge in water treatment. Magnetic nanocomposites (MNCs) based on Fe_3_O_4_ or NiFe_2_O_4_ cores functionalized with hydrophilic and charged polymer coatings represent a promising class of adsorbents for Cr^6+^ remediation. Their advantages include tunable surface chemistry, high adsorption capacity, and facile separation from treated water using an external magnetic field, as discussed in the previous section. Functional polymer capping significantly increases the number of accessible adsorption sites, improves colloidal stability in aqueous media, and enables efficient recovery and reuse of the adsorbents, which are essential features for practical water purification applications. Core–shell Fe_3_O_4_-based materials have been reported to efficiently adsorb Cr^6+^ from aqueous solutions with good reusability and stability^55^. In the present system, LQAA and CQAA networks containing sulfonate and carboxylate functional groups provide strong ionic interactions with Cr^6+^ oxyanions through electrostatic attraction and surface complexation. In contrast, neutral monomers mainly enhance water dispersibility but contribute less to binding strength^56^. The CQAA systems further enhance adsorption performance by forming a three-dimensional hydrogel network with increased porosity, improved mechanical stability, and facilitated ion diffusion, which is consistent with the behavior of magnetic hydrogel adsorbents reported for wastewater treatment applications^57^. Non-quaternized polymers or networks composed of non-charged comonomers typically rely on weaker interactions, such as hydrogen bonding or van der Waals forces, and therefore exhibit lower affinity toward anionic contaminants like Cr^6+^. In addition, the use of nickel ferrite (NiFe_2_O_4_) as the magnetic core provides higher chemical stability and, in some cases, superior magnetic responsiveness compared to Fe_3_O_4_. These properties are advantageous for repeated magnetic separation and regeneration cycles, offering a critical benefit for sustainable water purification systems^58^. Accordingly, systematic optimization of adsorption parameters including solution pH, contact time, initial Cr^6+^ concentration, and adsorbent dose was carried out for Fe_3_O_4_- and NiFe_2_O_4_-based MNCs capped with LQAA, CQAA, and CAA to enable meaningful comparison with previously reported systems.

#### Optimization of Cr(VI) adsorption conditions

- Effect of solution pH.

**Fig. 10:**
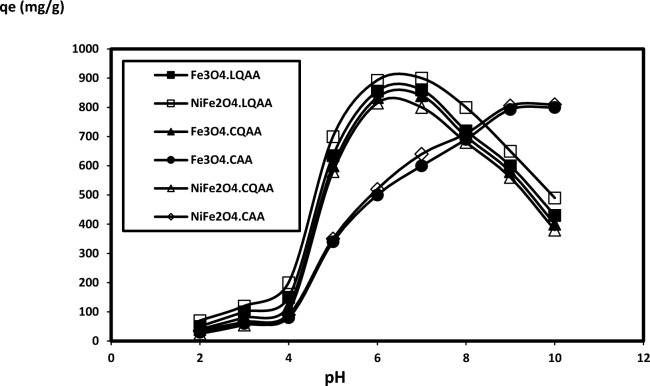
Effect of pH on adsorption capacity of aqueous solution Cr ^6+^ by MNCs.

Solution pH plays a crucial role in governing both Cr^6+^ speciation and the surface charge of the adsorbents, as illustrated in Fig. 10. The adsorption capacity increases sharply as the pH rises from 2 and reaches a maximum at pH 5–6, followed by a gradual decline at higher pH values. Under acidic conditions, Cr^6+^ predominantly exists as hydrochromate HCrO_4_^−^ and dichromate Cr_2_O₇^2^^−^ species, which strongly interact with positively charged quaternary ammonium groups in LQAA and CQAA via electrostatic attraction. Enhanced protonation of surface functional groups at low pH further promotes Cr^6+^ binding. The superior performance of CQAA-based MNCs at the optimal pH is attributed to the synergistic presence of quaternary ammonium and sulfonate groups, which provide strong electrostatic interactions along with hydrophilic pathways that facilitate Cr ion diffusion. At pH values above 7, the observed decrease in adsorption capacity is mainly due to increased competition from OH^−^ ions and reduced electrostatic attraction resulting from partial deprotonation of surface functional groups. Moreover, Cr^6+^ exists primarily as CrO_4_^2^^−^ under alkaline conditions, which experiences stronger electrostatic repulsion from negatively charged surfaces, particularly in CAA-based hydrogels. This pH-dependent adsorption behavior agrees well with previously reported Cr^6+^ adsorption systems based on quaternized polymers and magnetic composites^20,29,55^.

The ζ-potentials of the dispersed MNCs after Cr ion adsorption at pH 5.8, were measured and listed in Table 4. By comparing the surface charges of the MNCs before and after Cr adsorption, it observed that distinct charge modifications are observed. For LQAA and CQAA systems, ζ-potentials become more negative (e.g., LQAA–Fe_3_O_4_: + 8.9 mV at pH 6 before adsorption to − 31.2 mV after adsorption), indicating adsorption of anionic Cr^6+^ species (HCrO_4_^−^/Cr_2_O₇^2^^−^), which overcompensate the initial positive surface charge through electrostatic attraction and possible inner-sphere complexation. Conversely, for CAA systems, the magnitude of negative ζ-potential decreases after adsorption (e.g., − 30.6 mV to − 13.8 mV), suggesting partial charge neutralization via electrostatic interaction with protonated surface sites or reduction of Cr^6+^ to Cr^3+^ followed by surface complexation. The observed behavior aligns with established chromium speciation at pH ~ 5–6, where HCrO_4_^−^ predominates and electrostatic interactions strongly influence adsorption onto positively charged or functionalized iron oxide surfaces^59^. The results confirm that surface charge modulation by polymer architecture (linear vs. crosslinked; ammonium vs. acidic functionality) governs electrostatic interactions, stability, and chromium uptake efficiency in these magnetic nanocomposites.

(b) Effect of adsorbent dose.

**Fig. 11:**
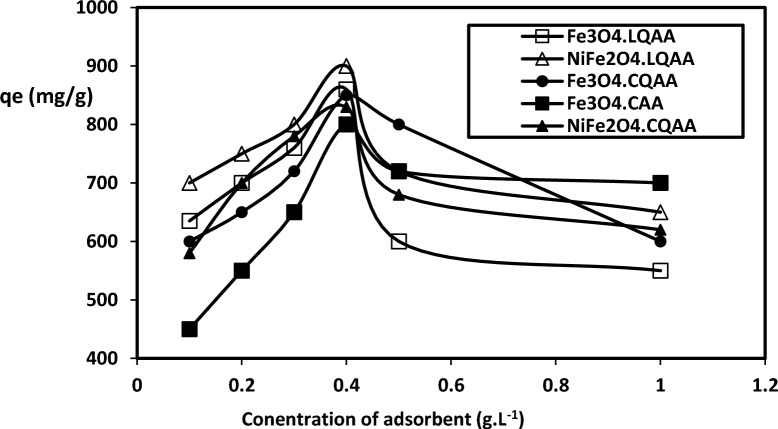
Effect of weights of MNCs on their adsorption capacities for Cr^6+^ aqueous solution.

The influence of adsorbent dose on Cr^6+^ adsorption capacity at the optimum pH is shown in Fig. 11. Increasing the MNCs dose from 0.02 to 0.04 g in 100 mL of Cr aqueous solution (0.2–0.4 g L^−1^) leads to a marked increase in the equilibrium adsorption capacity (q_e_), owing to the availability of a larger number of active sites and increased effective surface area. However, further increases in adsorbent dose result in a gradual decrease in q_e_. This decline can be attributed to aggregation of nanoparticles at higher dosages, which reduces the effective surface area and causes partial overlap or shielding of adsorption sites. In addition, for a fixed Cr^6+^ concentration, the number of Cr ions becomes insufficient to occupy the increasing number of available sites, leading to a lower adsorption capacity per unit mass. The observed optimal adsorbent dose therefore represents a balance between maximizing adsorption efficiency and minimizing material consumption. Among all materials investigated, CQAA- and LQAA-capped MNCs consistently exhibit higher adsorption capacities, highlighting the crucial role of quaternized functional groups in enhancing Cr ions removal through electrostatic attraction and ion-exchange mechanisms.

(c) Effect of initial Cr ions concentration.

**Fig. 12:**
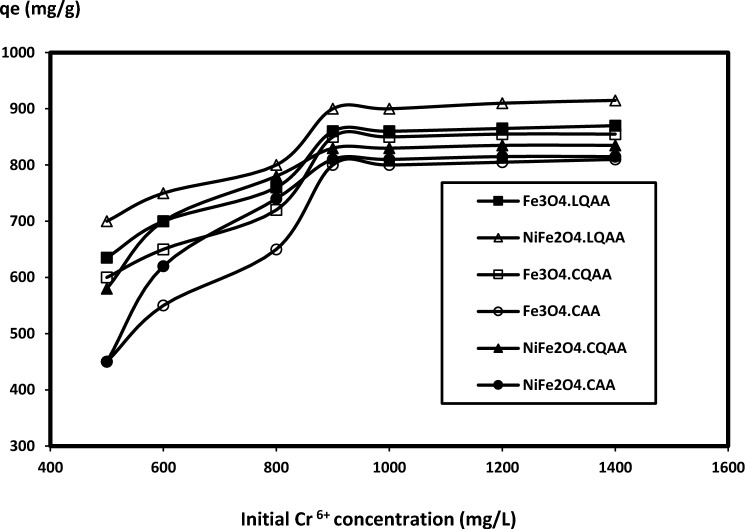
Effect of Initial Cr^6+^ concentrations on adsorption capacity of MNCs.

The effect of initial Cr(VI) concentration on the adsorption capacity (q_e_) of Fe_3_O_4_- and NiFe_2_O_4_-based MNCs capped with LQAA, CQAA, and CAA is presented in Fig. 12. The experiments were conducted at the optimum pH and adsorbent dose (0.01–0.02 g) determined in the previous sections, at room temperature. A pronounced increase in q_e_ is observed as the initial Cr ions concentration increases from 400 to approximately 800–900 ppm, followed by a plateau at higher concentrations. This behavior reflects a higher driving force for mass transfer at elevated solute concentrations, resulting from an increased concentration gradient between the solution and the adsorbent surface. At low Cr ions concentrations, the number of available adsorption sites exceeds the number of Cr ions, leading to incomplete utilization of active sites. As the concentration increases, more Cr^6+^ oxyanions interact with quaternary ammonium groups in LQAA and CQAA and with protonated functional groups in CAA, resulting in enhanced adsorption. The saturation trend at higher concentrations indicates progressive occupation of adsorption sites and limited availability of binding centers. Notably, NiFe_2_O_4_-based nanocomposites consistently show slightly higher adsorption capacities than their Fe_3_O_4_ counterparts, which can be attributed to higher surface charge density, improved dispersion within the polymer matrix, and stronger metal–polymer interfacial interactions. Similar trends have been reported for Cr ions adsorption on functionalized magnetic nanomaterials.

(d) Effect of contact time.

**Fig. 13:**
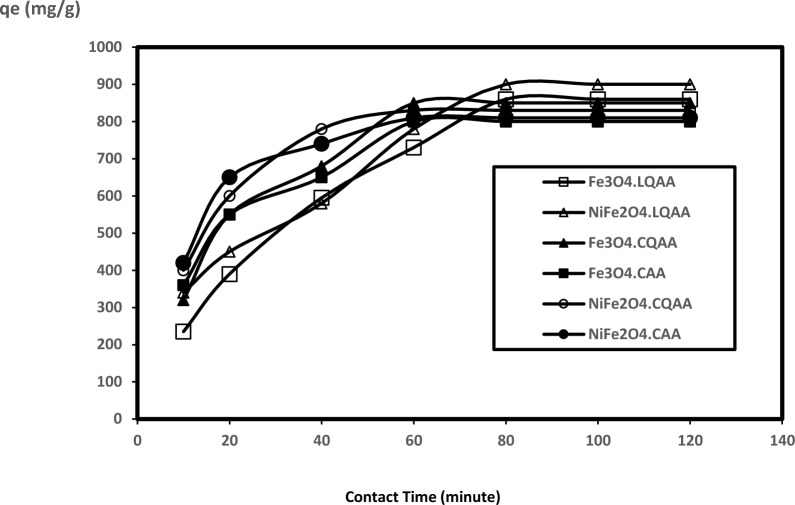
Effect of contact time on adsorption capacity of Cr^6+^ by MNCs.

The effect of contact time on Cr ions adsorption by the synthesized MNCs was evaluated at the optimum pH and adsorbent dose, and the results are shown in Fig. 13. A rapid initial adsorption occurs within the first 20–40 min, followed by a slower approach to equilibrium within 60–90 min. The fast initial uptake is attributed to the abundance of available active sites on the external surface of the adsorbents, enabling rapid electrostatic binding and surface complexation. As the adsorption progresses, intraparticle diffusion and gradual saturation of adsorption sites become the rate-limiting steps, leading to a slower adsorption stage. The relatively short equilibrium time indicates a strong affinity between Cr ions and the functionalized MNCs, particularly those incorporating LQAA and CQAA. The three-dimensional crosslinked structures of CQAA and CAA hydrogels facilitate efficient ion transport while maintaining structural stability, resulting in high adsorption rates. NiFe_2_O_4_-based composites again exhibit faster adsorption kinetics than Fe_3_O_4_-based systems, likely due to enhanced dispersion and improved accessibility of adsorption sites. Such rapid adsorption behavior is highly advantageous for practical wastewater treatment applications.

### Adsorption and reduction of Cr ions on MNCs surfaces

The speciation and quantification of chromium ions were systematically determined using complementary UV–visible spectroscopy and ICP–OES techniques as reported in the experimental section. The optimum adsorption conditions pH (5.8), contact time (2 h), weight of MNCs (0.1 g) and Cr concentration (1000 mg L^−1^) were applied to determine the adsorbed and remained Cr ions in water. Hexavalent chromium (Cr^6+^) was selectively analyzed by UV–visible spectroscopy in the presence of 1,5-diphenylcarbazide, which forms a characteristic colored complex measured at 554 nm, enabling accurate determination of the residual Cr^6+^ concentration. The calibration and UV–visible curves of the remained Cr^6+^ after adsorption on MNCs surfaces under optimum conditions were represented in supplementary file Fig. S3a,b. The remained Cr^6+^ concentrations were calculated from linear relation obtained from calibration curve (A = 0.0023[Cr^6+^ concentration]) after determine the absorbance (A) of aqueous solution that summarized in Table 5. In parallel, ICP–OES was employed to quantify the total chromium content and distinguish between Cr^6+^ and Cr^3+^ species at equilibrium, allowing indirect evaluation of reduction processes and total chromium uptake. The combined approach provides reliable insight into both adsorption and redox behavior of the MNCs. The data in Table 5 reveal that LQAA-based nanocomposites exhibit superior chromium removal efficiencies, with total adsorption reaching 85.3% and 89.6% for Fe_3_O_4_ and NiFe_2_O_4_, respectively, accompanied by relatively low residual Cr^6+^ fractions (11.6% and 10.0%). Notably, NiFe_2_O_4_.LQAA shows minimal formation of Cr^3+^ (4.23 mg L^−1^), indicating that adsorption is the dominant mechanism with limited reduction. In contrast, CQAA-modified samples demonstrate moderate performance, where Fe_3_O_4_.CQAA shows increased Cr^3+^ formation (50.40 mg L^−1^), suggesting a coupled adsorption–reduction mechanism, while NiFe_2_O_4_.CQAA exhibits the lowest adsorption efficiency (77.0%) and the highest residual Cr^6+^ (16.6%). For CAA systems, Fe_3_O_4_.CAA displays significant Cr^6+^ reduction (60.35 mg L^−1^ Cr^3+^), though with moderate total removal (79.5%), whereas NiFe_2_O_4_.CAA shows high residual Cr^6+^ (19.1%) and negligible Cr^3+^ formation, indicating weak interaction with chromium species. The results confirm that both the ferrite type and polymer functionality strongly influence chromium removal performance, with LQAA-functionalized MNCs providing the most efficient adsorption and CQAA/CAA systems promoting partial reduction of Cr^6+^ to Cr^3+^. These data confirm that the presence of Fe^2+^ in the magnetite facilitates the reduction of Cr^6+^ to Cr^3+^ than NiF_2_O_4_ MNCs as well as the ability of protic PIL based on LQAA and CQAA to complete hexavalent chromium ions to trivalent reduction reaction^60^.

**Table 5:** The remained Cr^6+^ and total Cr ions concentrations after adsorption of their aqueous solutions in the presence of MNCs at optimum adsorption conditions.

% Cr ions			Total concentration of remained Cr ions at equilibrium determined by ICP-OES (mg L^-1^)		UV–visible		Ferrite type	Capping PILs
Remained Cr^6+^	Remained Cr^6+^	Total adsorbed	Cr^3+^	Cr^6+^	Remained concentration Cr^6+^ mg L^−1^	Absorbance at 540 nm		
3.1	11.6	85.3	30.89 ± 0.23	116.50 ± 0.5	116.50 ± 1.43	0.26795	Fe_3_O_4_	LQAA
0.4	10.0	89.6	4.23 ± 0.12	100.41 ± 0.03	100.41 ± 0.15	0.22094	Ni.Fe_2_O_4_	
5.1	10.1	84.8	50.40 ± 0.31	107.32 ± 0.02	107.32 ± 0.42	0.24684	Fe_3_O_4_	CQAA
6.4	16.6	77.0	6.12 ± 0.15	166.56 ± 0.23	166.56 ± 0.31	0.383088	Ni.Fe_2_O_4_	
6.0	14.5	79.5	60.35 ± 0.76	145.43 ± 0.28	145.43 ± 0.87	0.104489	Fe_3_O_4_	CAA
0.2	19.1	80.7	1.62 ± 0.43	190.01 ± 0.26	190.36 ± 1.09	0.204828	Ni.Fe_2_O_4_	

FTIR spectra of adsorbed Cr ions on the Cr^6+^, Cr=O asymmetric absorption peak, and Cr−O stretching vibration peak in chromate ions appeared near at 767 cm^−1^ and 943 cm^−1^, respectively, which proved that the N^+^–C–C–N^+^ structure successfully combines with CrO_4_^2−^.The presence and extent of Cr^6+^ were evaluated by monitoring characteristic vibrational bands corresponding to chromate and dichromate ions. Typically, Cr species exhibit strong absorption bands in the range of 900–1000 cm^−1^, attributed to the asymmetric stretching vibration of Cr = O bonds (ν_3_), along with additional bands related to Cr–O stretching modes depending on the coordination environment and pH conditions. Upon interaction with the host material, noticeable shifts in the position and/or intensity of these bands were observed, indicating coordination, electrostatic interaction, or partial reduction processes. Changes in functional group regions (e.g., –OH, –NH, or –COO^−^) represented in Fig. 2 and Table 1 of MNCs before Cr adsorption further supported the involvement of active sites in binding Cr^6+^.

**Fig. 14:**
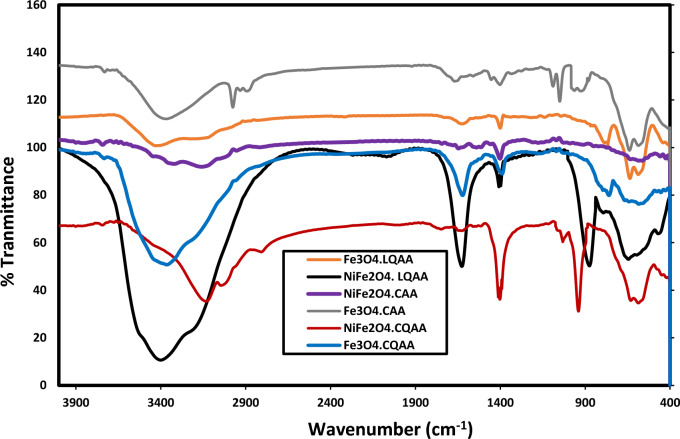
FTIR spectra of the prepared MNCs after Cr ions adsorption.

The FTIR spectrum of Fe_3_O_4_. LQAA before Cr adsorption (Fig. 2) exhibits characteristic bands at 3133–3077 cm^−1^ (O–H/N–H stretching), 1735 cm^−1^ (C=O), 1644 and 1546 cm^−1^ (asymmetric and symmetric COO^−^/amide vibrations), and 1193–1038 cm^−1^ corresponding to S=O stretching of sulfonate groups, confirming successful coating of Fe_3_O_4_ with the LQAA. After Cr ions adsorption FTIR spectra of the prepared MNCs (Fig. 14) show the O–H/N–H band significantly broadens and shifts (centered around 3399 cm^−1^), indicating involvement of hydroxyl and ammonium groups in chromium binding. The COO^−^ band shifts from 1633 to 1626 cm^−1^ with noticeable intensity changes at 1402 cm^−1^, and modifications in the sulfonate region further confirm participation of –SO_3_^−^ groups in adsorption. The Ni–O/Fe–O band remains present (566 cm^−1^), demonstrating structural stability of the magnetic core. After Cr ions adsorption (Fig. 14), noticeable shifts and intensity changes in the broad O–H/N–H band (3428 cm^−1^) and the COO^−^ vibrations (1642 and 1400 cm^−1^) are observed, indicating involvement of carboxylate and ammonium functionalities in metal binding. Modifications in the sulfonate region further suggest participation of –SO_3_^−^ groups. The persistence of the Fe–O band confirms structural stability of the magnetic core. Overall, the spectral changes support adsorption through combined electrostatic interactions and surface complexation mechanisms. The FTIR spectrum of NiF_2_O_4_.LQAA (Fig. 14) shows, the O–H/N–H band shifts and broadens (3399 cm^−1^), indicating involvement of hydroxyl and ammonium functionalities in binding after Cr ions adsorption. Changes in the COO^−^ (1623 and 1402 cm^−1^) and sulfonate regions further suggest coordination and electrostatic interactions with chromate species. By comparing FTIR spectrum of Fe_3_O_4_. LQAA and Fe_3_O_4_. CQAA (Fig. 14), it was found that the MNCs capped with CQAA exhibits smaller spectral shifts, suggesting that crosslinking restricts chain mobility and limits functional group accessibility, leading to more surface-confined adsorption behavior^61^. Moreover, it suggested that the Cr ion adsorption occurs through combined electrostatic attraction with protonated ammonium groups and surface complexation with COO^−^ and –SO_3_^−^ functionalities, but in a more surface-confined manner than in the LQAA system. Consequently, the FTIR spectra of MNCs after Cr ions adsorption arranged the order of adsorption as: LQAA > CQAA > CAA, highlighting the critical role of quaternization and polymer architecture in enhancing Cr^6+^ uptake.

#### Adsorption isotherms

Adsorption is usually described through isotherms, that is, the amount of adsorbate on the adsorbent as a function of its pressure (if gas) or concentration (if liquid) at constant temperature. Adsorption isotherm is important to describe how adsorbates interact with adsorbents and so it is important in optimizing the use of adsorbents. Three are most common isotherm equations namely, Langmuir, and Freundlich and were tested here^62^. The basic assumption of the Langmuir model is that adsorption takes place at specific homogeneous sites within the adsorbent. It is further assumed that once a Cr^6+^ molecule occupies a site, no further adsorption can take place at that site^63^. Moreover, the surface of the adsorbent is uniform, that is, all the adsorption sites are equivalent (only a monolayer is formed). Also, adsorbed molecules do not interact and all adsorption occurs through the same mechanism. The Langmuir equation can be written as follows^62^:

5 $$q_{e} = [({\mathrm{Q}}_{{{\mathrm{max}}}} K_{l} C_{e} )/({1} + K_{l} C_{e} )]$$

where q_e_ is the amount of dye adsorbed on nanocomposites at equilibrium (mg/g), Q_0_ is the maximum adsorption capacity (mg/g), K_L_ is the Langmuir constant, and C_e_ is the concentration of dye solution at equilibrium (mg/L). The linear form of Langmuir equation is:

6 $$C_{e} /q_{e} = [({1}/{\mathrm{Q}}_{{{\mathrm{max}}}} Kl) \, + \, (C_{e} /{\mathrm{Q}}_{{{\mathrm{max}}}} )$$

The monolayer adsorption capacity, Q and the Langmuir constant K_l_ can be obtained from the linear plot of C_e_/q_e_ against C_e_.

The Freundlich equation is basically empirical equation and employed to describe heterogeneous systems. The isotherm assumes that adsorbent surface sites have a spectrum of different binding energies. The Freundlich equation is given by:

7 $$q_{e} = K_{{\mathrm{f}}} C_{e}^{{{1}/{\mathrm{n}}}}$$

where K_F_ is the Freundlich constant, which is a comparative measure of the adsorption capacity of the adsorbent and n is an empirical constant which gives valuable information about the isotherm shape. 1/n values indicate the type of isotherm to be irreversible (1/n = 0), favorable (0 < 1/n < 1) and unfavorable (1/n > 1). The Freundlich parameters can be obtained from the following linearized equation:

8 $$\ln q_{{\mathrm{e}}} = [(\ln K_{{\mathrm{f}}} ) + {1}/{\mathrm{n}}(\ln C_{e} )]$$

By linear plotting ln *q*_*e*_ as the function of ln *Ce*, the values of *K*_*f*_ and *n* can be obtained from the *slope* and the intercept of the plot. Figures S4 and S5 present the linearized Langmuir and Freundlich isotherms for Cr(VI) adsorption onto the prepared MNCs. The Langmuir isotherm parameters for Cr^6+^ adsorption on Fe_3_O_4_^−^ and NiFe_2_O_4_-based nanocomposites capped with LQAA, CQAA, and CAA are summarized in Table 6. The Langmuir constant (K_1_, L mg^−1^) and the maximum adsorption capacity (Q_max_, mg g^−1^) were calculated from the intercept (1/Q_max_K_1_) and slope (1/Q_e,exp_) of the linear Ce/qe versus Ce plots, respectively. All systems exhibit very high correlation coefficients (R^2^ = 0.9954–0.9999), indicating that Cr^6+^ adsorption follows a Langmuir-type mechanism dominated by monolayer coverage on a homogeneous surface with a finite number of identical active sites. LQAA-capped nanocomposites show high experimental adsorption capacities (Q_e,exp_ 860–900 mg g^−1^), in excellent agreement with their fitted Q_max_ values, confirming efficient utilization of available adsorption sites. Notably, NiFe_2_O_4_.LQAA exhibits a slightly higher Q_max_ (900.1 mg g^−1^) than Fe_3_O_4_.LQAA (860.3 mg g^−1^), which can be attributed to nickel substitution enhancing surface charge density and electrostatic interactions with anionic Cr^6+^ species, together with the high accessibility of quaternary ammonium groups along the LPIL chains. CQAA-capped nanocomposites show slightly lower Q_e,exp_ values (815–840 mg g^−1^) compared to their LQAA counterparts. This decrease is attributed to the crosslinked polymer network, which improves structural stability but partially restricts chain mobility and limits accessibility of quaternized adsorption sites. Nevertheless, the close agreement between Q_e,exp_ and Q_max_, along with high R^2^ values, confirms uniform and efficient adsorption. The higher K_1_ values observed for CQAA systems indicate stronger Cr^6+^ binding affinity, likely arising from cooperative electrostatic interactions within the crosslinked matrix. CAA-based nanocomposites exhibit distinct adsorption behavior. Fe_3_O_4_.CAA shows an exceptionally high fitted Q_max_ (1250 mg g^−1^), far exceeding its experimental capacity, suggesting the presence of a large number of theoretical adsorption sites contributed by abundant carboxylate and sulfonate groups. However, steric hindrance and diffusion limitations within the highly crosslinked CAA network likely prevent full utilization of these sites. In contrast, NiFe_2_O_4_.CAA displays a Q_max_ value closer to Q_e,exp_ and a markedly higher K_1_ (0.2667 L mg^−1^), indicating stronger adsorption affinity but fewer accessible sites, consistent with the formation of a compact adsorption layer dominated by strong electrostatic interactions. Overall, the effective experimental adsorption capacity follows the trend LQAA ≥ CQAA ≥ CAA, highlighting the importance of polymer chain flexibility and accessibility of quaternary ammonium groups, while higher K_1_ values for CAA systems reflect stronger Cr^6+^–adsorbent interactions.

**Table 6:** Langmuir isotherm parameters and correlation coefficient for adsorption of Cr^6+^ by MNCs at optimum conditions and 25 °C.

Sample code	Q_e,exp_ (mg g^−1^)	Q_max_ (mg g^−1^)	*K*_L_ (mg g^−1^)	R^2^
Fe_3_O_4_.LQAA	860.3 ± 2.1	862.1 ± 1.8	0.1009 ± 0.001	0.9999
NiFe_2_O_4_.LQAA	900.1 ± 4.3	909.1 ± 2.3	0.1425 ± 0.003	0.9999
Fe_3_O_4_.CQAA	840.4 ± 2.5	769.2 ± 1.3	0.0731 ± 0.002	0.9957
NiFe_2_O_4_.CQAA	815.3 ± 1.8	833.3 ± 1.7	0.0646 ± 0.003	0.9954
Fe_3_O_4_.CAA	800.1 ± 1.2	1250 ± 3.4	0.1695 ± 0.008	0.9995
NiFe_2_O_4_.CAA	810.3 ± 1.3	769.2 ± 1.1	0.2667 ± 0.009	0.9997

The Freundlich isotherm parameters for Cr^6+^ adsorption onto the MNCs are listed in Table 7. The Freundlich constants Kf (mg g^−1^) and 1/n were obtained from the intercept (ln Kf) and slope (1/n) of the linear plots of ln qe versus ln Ce, as described in the Supplementary Information. The Freundlich model accounts for adsorption on heterogeneous surfaces with non-uniform energy distribution and provides insight into adsorption intensity and surface heterogeneity. For all nanocomposites, the 1/n values are below unity (0.0554–0.2032), indicating favorable adsorption over the investigated concentration range. LQAA-capped systems exhibit moderate to high Kf values (14.04–15.94 mg g^−1^), confirming their strong affinity toward Cr^6+^ ions. NiFe_2_O_4_.LQAA shows a higher Kf and lower 1/n than Fe_3_O_4_.LQAA, reflecting enhanced adsorption intensity and a more energetically favorable surface, likely due to increased surface charge density resulting from nickel incorporation. CQAA-capped nanocomposites display lower Kf values (11.71–13.60 mg g^−1^) and slightly higher 1/n values, suggesting reduced adsorption intensity and increased surface heterogeneity caused by polymer crosslinking and restricted functional group accessibility, consistent with their lower Freundlich correlation coefficients (R^2^ ≈ 0.8605). CAA-based nanocomposites exhibit the highest adsorption affinity, as evidenced by the highest Kf values (16.43–17.60 mg g^−1^) and the lowest 1/n values, particularly for NiFe_2_O_4_.CAA (1/n = 0.0949). This behavior is attributed to the high density of carboxylate and sulfonate groups within the CAA network, which interact strongly with Cr^6+^ ions through electrostatic attraction and complexation. The high correlation coefficients (R^2^ = 0.9471–0.9778) confirm the suitability of the Freundlich model for these heterogeneous surfaces. Overall, the isotherm analysis demonstrates that polymer architecture and ferrite composition play decisive roles in governing Cr^6+^ adsorption behavior. While LQAA-based systems offer an optimal balance between adsorption capacity and surface homogeneity, CAA-based nanocomposites exhibit the strongest adsorption affinity due to their multifunctional acidic groups. The combined Langmuir and Freundlich results confirm efficient, favorable, and tunable Cr^6+^ adsorption on the prepared magnetic nanocomposites.

**Table 7:** Freundlich isotherm parameters and correlation coefficient for adsorption of Cr^6+^ by MNCs at optimium conditions and 25 °C.

Sample code	Q_e,exp_ (mg g^−1^)	1/n	K_F_ (L g^−1^)	R^2^
Fe_3_O_4_.LQAA	860.3 ± 2.1	0.0888 ± 0.003	14.04 ± 0.02	0.9471
NiFe_2_O_4_.LQAA	900.1 ± 4.3	0.0554 ± 0.001	15.94 ± 0.05	0.9740
Fe_3_O_4_.CQAA	840.4 ± 2.5	0.2032 ± 0.040	11.71 ± 0.03	0.8605
NiFe_2_O_4_.CQAA	815.3 ± 1.8	0.1202 ± 0.008	13.60 ± 0.01	0.8402
Fe_3_O_4_.CAA	800.1 ± 1.2	0.1559 ± 0.009	17.60 ± 0.06	0.8844
NiFe_2_O_4_.CAA	810.3 ± 1.3	0.0949 ± 0.003	16.43 ± 0.05	0.9778

#### Adsorption kinetics

**Fig. 15:**
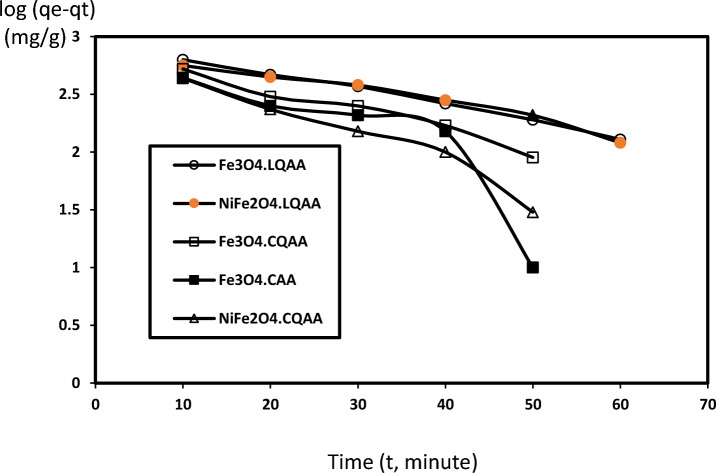
Kinetic study pseudo-first order for adsorption of Cr ^6+^ by MNCs.

**Fig. 16:**
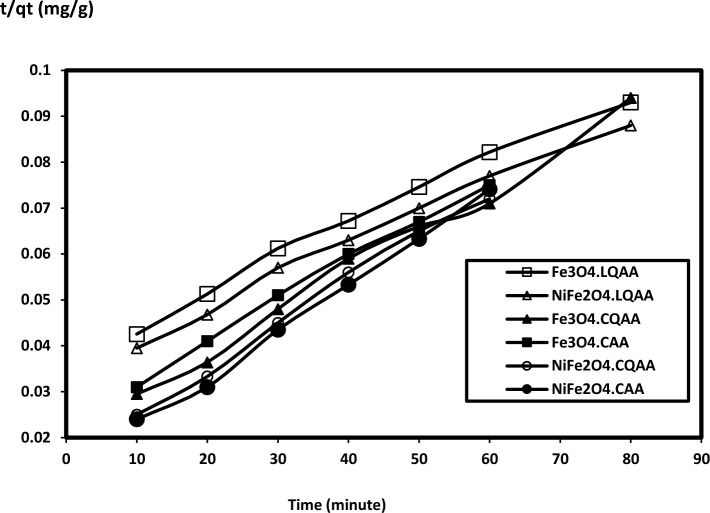
Kinetic study pseudo-second order for adsorption of Cr ^6+^ by MNCs.

The adsorption kinetics of Cr^6+^ onto Fe_3_O_4_ and NiFe_2_O_4_ nanocomposites functionalized with LQAA, crosslinked quaternized CQAA, and CAA were evaluated using pseudo-first-order (PFO) and pseudo-second-order (PSO) kinetic models. The linearized form of the PFO model, proposed by Lagergren, is given by:

9 $${\mathrm{Log}}\left( {{\mathrm{q}}_{{\mathrm{e}}} - {\mathrm{q}}_{{\mathrm{t}}} } \right) = {\text{ logq}}_{{\mathrm{e}}} - {\text{ K}}_{{1}} {\mathrm{t}}/{2}.{3}0{3}$$

where q_e_ and q_t_ (mg g^−1^) represent the adsorption capacities at equilibrium and at time t, respectively, and k1 (min^−1^) is the PFO rate constant. Linear plots of log (q_e_ − q_t_) versus time for Cr^6+^ adsorption onto the investigated nanocomposites are shown in Fig. 15. The corresponding kinetic parameters and correlation coefficients are summarized in Table 8. Although relatively high correlation coefficients (R^2^ ≈ 0.95–0.99) were obtained for LQAA- and CQAA-based nanocomposites, noticeable discrepancies between calculated and experimental equilibrium capacities. The PSO kinetics may be expressed in a linear form as:

10 $${\mathrm{t}}/{\mathrm{q}}_{{\mathrm{t}}} = \, [\left( {{1}/{\mathrm{K}}_{{2}} {\mathrm{q}}_{{\mathrm{e}}}^{{2}} + \, \left( {{\mathrm{t}}/{\mathrm{q}}_{{\mathrm{t}}} } \right)} \right]$$

where k2 (g mg^−1^ min^−1^) is the PSO rate constant. Plots of t/q_t_ versus t (Fig. 16) were used to determine q_e_ and k2, and the derived parameters are listed in Table 9. The PSO model provides an excellent fit for all nanocomposites, with correlation coefficients exceeding 0.99 in most cases and close agreement between calculated and experimental adsorption capacities. These results indicate that chemisorption is the rate-limiting step, involving electron sharing or exchange between Cr^6+^ species and surface functional groups. The adsorption process is governed primarily by interactions between Cr^6+^ ions and quaternary ammonium groups in LQAA- and CQAA-based systems, as well as carboxylate and sulfonate groups in CAA-based nanocomposites. The PSO rate constants further highlight the influence of polymer architecture and ferrite composition. CAA-based nanocomposites exhibit the highest k2 values, particularly NiFe_2_O_4_.CAA (8.03 × 10^−5^ g mg^−1^ min^−1^), reflecting rapid adsorption due to the high density of ionizable functional groups and strong electrostatic attraction toward Cr^6+^ ions. Similarly, CQAA-based nanocomposites show higher k2 values than their LQAA counterparts, indicating enhanced adsorption kinetics arising from cooperative interactions within the crosslinked polymer network. Overall, NiFe_2_O_4_-based nanocomposites display superior kinetic performance compared to Fe_3_O_4_ analogues, likely due to increased surface reactivity associated with nickel substitution in the ferrite lattice. Although LQAA-based systems exhibit comparatively lower rate constants, they still maintain high adsorption capacities owing to the flexibility and accessibility of LQAA chains.

**Table 8:** PFO kinetic parameters for adsorption of 1000 ppm of Cr^6+^ by MNCs.

Sample code	Q_e,exp_ (mg g^-1^)	q_e,cal_	*K*1	R^2^
Fe_3_O_4_.LQAA	860.3 ± 2.1	895.4 ± 3.5	0.0313	0.9942
NiFe_2_O_4_.LQAA	900.1 ± 4.3	829.7 ± 3.1	0.0295	0.9582
Fe_3_O_4_.CQAA	840.4 ± 2.5	778.75 ± 1.8	0.041	0.9703
NiFe_2_O_4_.CQAA	815.3 ± 1.8	872.87 ± 2.9	0.062	0.9527
Fe_3_O_4_.CAA	800.1 ± 1.2	1449.44 ± 4.6	0.081	0.7457
NiFe_2_O_4_.CAA	810.3 ± 1.3	785.236 ± 2.6	0.0693	0.9673

**Table 9:** PSO order kinetic parameters for adsorption of for adsorption of 1000 ppm of Cr^6+^ by MNCs at optimum conditions.

Sample code	Q_e,exp_	q_e,cal_	k_2_* 10^5^	R^2^
	(mg g^-1^)		(g mg^−1^ min^−1^)	
Fe_3_O_4_.LQAA	860.3 ± 2.1	1428.6 ± 5.3	1.306	0.992
NiFe_2_O_4_.LQAA	900.1 ± 4.3	1428.6 ± 5.2	1.440	0.9915
Fe_3_O_4_.CQAA	840.4 ± 2.5	1111.1 ± 4.8	4.050	0.9913
NiFe_2_O_4_.CQAA	815.3 ± 1.8	1000.1 ± 3.9	6.54	0.9943
Fe_3_O_4_.CAA	800.1 ± 1.2	1111.1 ± 4.6	3.45	0.9953
NiFe_2_O_4_.CAA	810.3 ± 1.3	1000.3 ± 3.2	8.03	0.9969

### Adsorption mechanism

**Fig. 17:**
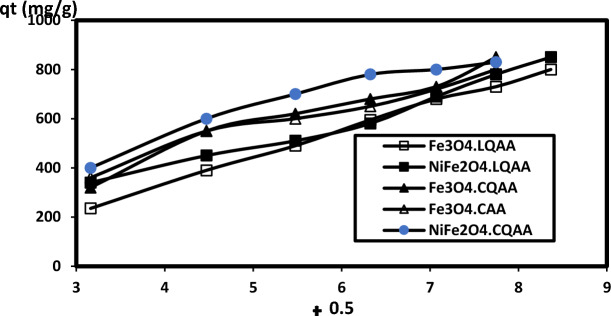
Intra-particle diffusion plots for adsorption of Cr^6+^ on MNCs.

To elucidate the adsorption mechanism and identify the rate-controlling steps governing Cr^6+^ uptake, the kinetic data were analyzed using the Weber–Morris interparticle diffusion model and the Boyd model. The interparticle diffusion model is expressed as:

11 $$q_{t} = K_{p} \cdot t^{{0.{5}}} + C$$

where q_t_ (mg g^−1^) is the amount of Cr(VI) adsorbed at time t, k_p_ (mg g^−1^ min^−0.5^) is the intraparticle diffusion rate constant, and C is the intercept related to boundary layer thickness. The values of kp and C were obtained from the slope and intercept of the linear plots of qt versus t^0.5^, as shown in Fig. 17, and are summarized in Table 10. If intraparticle diffusion were the sole rate-limiting step, the plots would be linear and pass through the origin. However, although good linearity was observed for all samples, none of the plots passed through the origin (C ≠ 0), indicating that intraparticle diffusion is not the only rate-controlling mechanism. The intercept values reflect the contribution of surface adsorption; higher C values correspond to a greater boundary layer effect during the initial adsorption stage. The calculated kp values range from 79.30 to 108.4 mg g^−1^ min^−0.5^, indicating rapid Cr^6+^ uptake for all MNCs. Fe_3_O_4_.LQAA (108.4 mg g^−1^ min^−0.5^) and Fe_3_O_4_.CQAA (104.19 mg g^−1^ min^−0.5^) exhibit the highest diffusion rates, which can be attributed to the high surface activity and abundant functional groups introduced by QAA-based modification. In contrast, NiFe_2_O_4_.CAA and NiFe_2_O_4_.CQAA show relatively large C values (232.29 and 156.55, respectively), suggesting a pronounced boundary layer effect and a dominant contribution of surface adsorption at the early stages. The much lower C value observed for NiFe_2_O_4_.LQAA (3.33) implies reduced external mass-transfer resistance and a stronger contribution of intraparticle diffusion. To further distinguish between film diffusion and intraparticle diffusion, the Boyd model was applied. The Boyd equation is given as:

12 $${\mathrm{F}} = { 1} - \, \left[ {\left( {{6}/\pi^{{2}} } \right){\mathrm{e}}_{{\mathrm{t}}}^{{({1} - \beta )}} } \right]$$

**Table 10:** Intraparticle diffusion and Boyd parameters for adsorption of 1000 ppm of Cr^6+^ by MNCs at optimum conditions.

Intraparticle diffusion model				Boyd model	
Sample	K (mg g^-1^ min^−0.5^ )	C	R^2^	Intercept	R^2^
Fe_3_O_4_.LQAA	108.4 ± 1.2	100.01 ± 0.8	0.9979	− 0.6626	0.9931
NiFe_2_O_4_.LQAA	98.03 ± 0.8	3.33 ± 0.03	0.9771	− 0.9327	0.9096
Fe_3_O_4_.CQAA	104.19 ± 2.1	30.22 ± 0.04	0.9605	0	0.9961
NiFe_2_O_4_.CQAA	92.57 ± 0.4	156.55 ± 0.23	0.9387	− 0.5570	0.991
Fe_3_O_4_.CAA	88.19 ± 0.3	109.86 ± 0.89	0.9697	− 0.2475	0.9688
NiFe_2_O_4_.CAA	79.30 ± 0.2	232.29 ± 1.23	0.8969	− 0.4279	0.9545

**Fig. 18:**
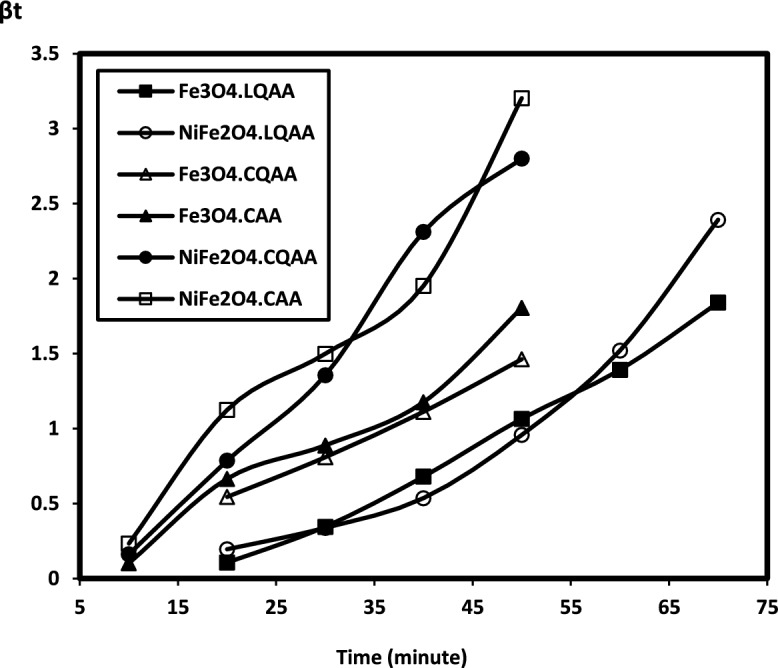
Boyd plot for adsorption of Cr^6+^ on MNCs.

This equation can be arranged as:

13 $${\rm B}_{t} = - 0.{4977} - {\mathrm{ln}}({1} - {\mathrm{F}})$$

where F is the fractional attainment of equilibrium (F = q_t/q_e), and Bt is a function of time. The plots of Bt versus t are shown in Fig. 18. If the Boyd plots are linear and pass through the origin, intraparticle diffusion controls the adsorption rate; otherwise, film diffusion is significant. For all investigated MNCs, the Boyd plots are linear but do not pass through the origin, indicating that external mass transfer (film diffusion) governs the initial adsorption stage, followed by intraparticle diffusion at later times. The high correlation coefficients (R^2^ = 0.9096–0.9961) confirm the reliability of the model fitting. Fe_3_O_4_.CQAA and NiFe_2_O_4_.CQAA exhibit particularly high linearity (R^2^ = 0.9961 and 0.991, respectively), suggesting well-defined diffusion behavior, although boundary layer resistance remains non-negligible. Based on the combined intraparticle diffusion and Boyd model analyses, Cr^6+^ adsorption onto the prepared MNCs proceeds through a multi-step mechanism:

1. Rapid external diffusion of Cr(VI) species from the bulk solution to the adsorbent surface.
2. Surface adsorption driven by electrostatic attraction between negatively charged Cr(VI) species (HCrO_4_^−^/Cr_2_O₇^2^^−^) and positively charged functional groups on the adsorbent surface under optimum conditions.
3. Gradual intraparticle diffusion of Cr(VI) into the internal pores of the nanocomposites, contributing to the later stages of adsorption.

The enhanced performance of QAA-modified and Fe_3_O_4_-based materials highlights the role of surface functionalization and magnetic core composition in facilitating both surface interaction and diffusion processes.

The ζ-potential variations at pH 5.8 provide clear insight into the chromium adsorption mechanism. For LQAA- and CQAA-capped Fe_3_O_4_/NiFe_2_O_4_ nanocomposites, the surface is initially positive or near neutral due to protonated triethanolammonium and ≡M–OH_2_⁺ groups; after Cr adsorption, a pronounced shift toward negative ζ-potentials is observed, indicating charge overcompensation by adsorbed HCrO_4_^−^ species. This behavior confirms that electrostatic attraction is the dominant mechanism, likely accompanied by partial inner-sphere complexation through ligand exchange with surface hydroxyl groups. In contrast, CAA-capped systems remain negatively charged before adsorption and exhibit a reduction in negative ζ-potential magnitude after Cr uptake, suggesting surface charge neutralization rather than reversal. Since electrostatic attraction is unfavorable in this case, the adsorption is attributed primarily to specific surface complexation and possible redox-assisted binding (Cr(VI) reduction to Cr(III)) followed by coordination with carboxylate/sulfonate groups. Overall, the ζ-potential analysis demonstrates that polymer functionality governs whether chromium removal proceeds predominantly via electrostatic interaction or chemisorption-driven mechanisms.

#### Comparison of the adsorption capacity of some sorbents toward Cr ions

A comparison of the Cr ions adsorption capacities obtained in the present study with those of previously reported on PILs composites and magnetic adsorbents is summarized in Table 11. The comparison clearly demonstrates that the developed materials exhibit remarkably high adsorption capacities (800–950 mg g^−1^), significantly outperforming most magnetic adsorbents and PILs composites reported in the literature, thereby confirming the effectiveness of PIL-based hydrogel functionalization. Imidazolium ILs are also highly effective as extractants, adsorbents and membranes for Cr ions, whether utilized directly or integrated into various sorbents to create functionalized materials. The mesoporous crosslinked PILs based on dicationic monomer (*N*,*N*′-methylene-bis(1-(3-vinylimidazolium)) chloride with surface area 246.2 m^2^ g^-1^ as was used as adsorbents for hexavalent Cr ions and displayed a very high sorption capacity (328.2 mg g^−1^ at 25 °C)^24^. It was previously reported that the Cr ions were efficiently adsorbed from water via electrostatic attraction and reduction of Cr^6+^ to Cr^3+^mechanism using zero-valent iron with imidazolyl-based ILs^22^. It was noteworthy that ILs can serve as an efficacious reductant for the recycling of Fe(II)/Fe(III), thereby augmenting the sequential conversion of Cr^6+^ to Cr^3+^ on its surface. Cr ions removal efficiency was evident across pH 5.8, and after being stored under anoxic conditions for a duration of 60 days. Mesoporous hyper-crosslinked imidazolium PILs with surface area ranged from 191.5 to 843.4 m^2^ g^−1^ were used to remove Cr ions from water via ion-exchange mechanism with removal adsorption capacity 236.8 mg g^−1^ during 10–20 min^23^. The PIL gel based on crosslinked 1-allyl-3-vinylimidazolium chlorid PIL gel achieved Cr ion removal capacity 283.3 mg g^−1^ through anion exchange mechanism^64^. Stable functionalized membrane based on polymerizable imidazolium based PILs (Methyl-3-(4-vinylbenzyl)-1Himidazole-3-ium chloride) exhibited outstanding performance in recovering Cr ions 20 times from water solutions^65^**.** It was also reported that lowest to modest values are commonly attributed to the limited density of accessible functional groups and partial blockage of active sites within dense imidazolium PILs matrices^66–68^. Metal–organic frameworks (MOFs) based on UiO-66 composites encapsulated with divinyl imidazolium exhibited outstanding stability, superior uptake capacity (787 mg g^−1^, 5 times of UiO-66) within 1 min^69^. Carbon nanotube, graphene oxide, ferrite phases, capped with protic ILs or conducting polymers such as polypyrrole (PPy) and polyaniline (PANI), reported maximum adsorption capacities generally remain below 300 mg g^−1^^51,70^. Although these enhancements arise from synergistic interactions and increased surface heterogeneity, such systems often suffer from synthetic complexity, limited control over surface charge, and aggregation of nanofillers, which restrict their overall adsorption efficiency. In contrast, the exceptionally high Cr(VI) uptake observed for the PIL-capped magnetite and nickel ferrite nanocomposites in this work can be attributed to several synergistic structural and chemical factors. First, the PIL hydrogel matrices based on LQAA and CQAA introduce a high density of permanently charged cationic sites (–NH⁺ and –OH groups associated with the triethanolammonium moiety), which strongly promote electrostatic attraction toward anionic Cr(VI) species (HCrO_4_^−^ and Cr_2_O₇^2^^−^), particularly under acidic conditions. Second, the presence of sulfonate groups enhances hydrophilicity and swelling behavior, facilitating rapid ion diffusion into the hydrogel network and maximizing the utilization of internal adsorption sites. Moreover, the comparison between linear (LQAA) and crosslinked (CQAA) PIL systems highlights the importance of controlled crosslinking in balancing polymer flexibility, structural stability, and site accessibility. While linear PIL coatings provide high chain mobility and efficient ion transport, crosslinked networks suppress polymer dissolution and form stable three-dimensional porous architectures, improving adsorption capacity and reusability. The strong interfacial interaction between the PIL hydrogel shell and the magnetic cores (Fe_3_O_4_ or NiFe_2_O_4_) ensures uniform dispersion of active sites and prevents nanoparticle aggregation. Additionally, the magnetic cores contribute surface hydroxyl groups that may participate in hydrogen bonding or surface complexation with Cr(VI) species. Nickel ferrite-based systems, in particular, may offer higher surface basicity and stronger metal–anion interactions, further enhancing adsorption performance. Overall, compared with previously reported magnetic nanocomposite adsorbents summarized in Table 11, the LQAA- and CQAA-capped magnetite and nickel ferrite nanocomposites developed in this study represent a significant advancement in Cr(VI) adsorption. Their outstanding adsorption capacities, combined with magnetic separability, tunable polymer chemistry, and excellent structural stability, make these materials highly promising candidates for advanced water purification and heavy metal remediation applications.

**Table 11:** Adsorption comparison of MNCs capped with PILs and reported studies on nanocomposites adsorbents for Cr(VI).

S. No	Adsorbent material	q_max_ (mg g^-1^)	References
1	Zero-valent iron with imidazolyl-based ionic liquids	194.15	^22^
2	Hypercrosslinked mesoporous PILs	236.80	^23^
3	Mesoporous crosslinked (*N*,*N*′- methylene-bis(1-(3-vinylimidazolium)) chloride PILs	328.20	^24^
4	1-Allyl-3-vinylimidazolium chlorid PIL gel	283.30	^64^
5	Polymerizable imidazolium based PILs	89.16	^65^
6	Poly (3-ethyl-1- vinylimidazolium bis (trifluoromethanesulfonyl) imide)	17.90	^66^
7	Pulverized hydrogel based on 1-allyl-3-methylimidazolium chloride	74.50	^67^
8	1-Butyl-3-vinyl imidazolium chloride	l51.23	^68^
9	MOFs based on UiO-66 encapsulated with divinyl imidazolium	787.12	^69^
	Tetra *n*-heptylammonium bromide IL/carbon nanotube composite	85.83	^51^
11	Magnetic polypyrrole (PPy)-polyaniline (PANI)/iron oxide (Fe_3_O_4_) nanocomposite	303.2	^70^
9	MNCs capped with PILs	800–950	This study

#### Desorption and reusability

**Fig. 19:**
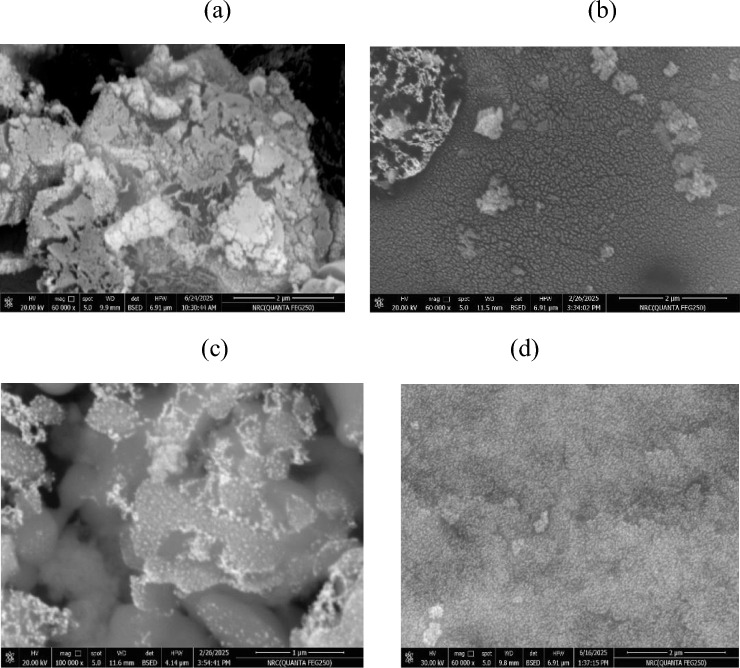
SEM micrographs of (**a**) NiFe_2_O_4_.CQAA, (**b**) Fe_3_O_4_.CQAA, (**c**) Fe_3_O_4_.CAA and (**d**) NiFe_2_O_4_.CAA after 4th regeneration cycle**.**

An effective adsorbent must exhibit not only high adsorption capacity but also good regeneration ability for repeated use. Alkaline solutions, particularly NaOH (0.1–0.3 M), are commonly employed as efficient eluents for Cr ions desorption from magnetic adsorbents^19^. Under alkaline conditions, electrostatic repulsion between anionic chromate species (HCrO_4_^−^, CrO_4_^2−^) and negatively charged adsorbent surfaces is enhanced, leading to efficient desorption, provided that the hydrogel backbone is stable in basic media. Because both Cr^6+^ speciation and adsorbent surface charge are strongly pH-dependent, desorption is most effective at high pH. After desorption, rinsing and re-equilibration at the working pH are required before subsequent adsorption cycles. Table 12 presents the adsorption capacities before and after regeneration using 0.3 M NaOH. Notably, the adsorption capacity increases after NaOH treatment, and the capacity in the second cycle is comparable to that of the first cycle, confirming that alkaline treatment is an effective regeneration strategy for Cr ions loaded MNCs. The enhanced adsorption capacity observed after regeneration may be attributed to partial rearrangement of the polymer network, which creates additional free volume and improves access to internal active sites during subsequent cycles^20^. Alternatively, thinning of the outer polymer layer during alkaline treatment may expose previously inaccessible adsorption sites. To evaluate reusability, four consecutive adsorption–desorption cycles were conducted at three different Cr ions concentrations. The regeneration efficiencies after four cycles are summarized in Table 12. All synthesized MNCs exhibit excellent regeneration behavior, retaining high adsorption efficiency over repeated cycles. For LQAA-based materials, both NiFe_2_O_4_·LQAA and Fe_3_O_4_·LQAA show outstanding stability. NiFe_2_O_4_·LQAA maintains nearly complete regeneration (100%) in the second cycle, with only a slight decrease to 99.4% and 98.8% in the third and fourth cycles, respectively. Similarly, Fe_3_O_4_·LQAA retains 99.9%, 98.7%, and 97.8% efficiency over successive cycles, indicating that Cr^6+^ adsorption is dominated by reversible electrostatic interactions. CQAA-based nanocomposites exhibit the highest regeneration efficiencies among all investigated materials. Both Fe_3_O_4_·CQAA and NiFe_2_O_4_·CQAA maintain almost unchanged adsorption performance, with regeneration efficiencies remaining close to 100% even after four cycles. This exceptional stability is attributed to the robust three-dimensional crosslinked CQAA network, which prevents polymer dissolution, structural collapse, or detachment from the magnetic core during regeneration. The crosslinked architecture also preserves the accessibility of cationic adsorption sites and minimizes irreversible Cr(VI) binding. In comparison, CAA-based materials show slightly lower regeneration efficiencies. Fe_3_O_4_·CAA exhibits a gradual decrease from 98.9% in the second cycle to 96.2% in the fourth cycle, while NiFe_2_O_4_·CAA decreases from 99.4% to 97.4%. This decline may result from weaker electrostatic interactions, partial saturation of adsorption sites, or gradual deterioration of functional groups during repeated cycling. Nevertheless, the CAA-based composites still demonstrate acceptable reusability, underscoring the stabilizing role of the magnetic core. Regarding the magnetic core, NiFe_2_O_4_-based nanocomposites generally display slightly higher regeneration efficiencies than their Fe_3_O_4_ counterparts. This behavior is likely due to the higher chemical stability and surface robustness of NiFe_2_O_4_, which helps maintain strong polymer–core interactions and reduces surface degradation during regeneration. The morphological stability of the MNCs after repeated adsorption–desorption cycles was evaluated by SEM analysis (Fig. 19). The SEM micrographs of NiFe_2_O_4_·CQAA, Fe_3_O_4_·CQAA, Fe_3_O_4_·CAA, and pristine NiFe_2_O_4_ after regeneration confirm the preservation of both the polymeric network and the magnetic core structure when compared with Fig. 5. The CQAA-based samples retain a compact yet porous morphology composed of interconnected domains, with no evidence of cracking, fragmentation, polymer peeling, or network collapse. The continuous coating around the magnetic cores indicates strong interfacial adhesion, consistent with the nearly constant regeneration efficiencies (> 99%). In contrast, Fe_3_O_4_·CAA exhibits a smoother and less interconnected morphology with slight surface densification after regeneration. Although no severe degradation is observed, this structural compaction may partially hinder mass transfer, explaining the modest decline in regeneration efficiency for CAA-based systems. The SEM image of NiFe_2_O_4_·CAA shows a uniform, densely packed granular morphology with no noticeable changes, confirming the intrinsic structural stability of the ferrite core. The absence of particle sintering or agglomeration further highlights the role of the polymer shell in maintaining dispersion and preventing magnetic aggregation. Overall, the SEM observations strongly support the regeneration results, confirming that the hydrogel networks particularly CQAA retain their structural integrity after repeated use. The lack of morphological degradation indicates that Cr(VI) adsorption occurs predominantly through reversible electrostatic interactions rather than irreversible binding or polymer degradation. Combined with high adsorption capacity and excellent reusability, these capped MNCs are well suited for long-term water treatment applications.

**Table 12:** Regeneration efficiencies of MNCs after repeated adsorption desorption cycles in the presence 1000 mg/L of Cr^6+^aqueous solution.

MNCs	Regeneration efficiencies in each cycle (%)		
	2nd	3rd	4th
NiFe_2_O_4_.LQAA	100 ± 0.3	99.4 ± 0.6	98.8 ± 0.9
Fe_3_O_4_.LQAA	99.9 ± 0.2	98.7 ± 0.8	97.8 ± 1.2
Fe_3_O_4_.CQAA	100 ± 0.1	99.9 ± 0.1	99.9 ± 0.1
NiFe_2_O_4_.CQAA	100 ± 0.1	99.9 ± 0.1	99.7 ± 0.2
Fe_3_O_4_.CAA	98.9 ± 0.2	97.6 ± 1.5	96.2 ± 0.9
NiFe_2_O_4_.CAA	99.4 ± 0.4	98.4 ± 1.8	97.4 ± 1.6

### Competitive adsorption of MNCs

MNCs based on Fe_3_O_4_ and NiFe_2_O_4_ are known to efficiently remove multiple heavy metal ions from aqueous media, with surface functionalization playing a key role in enhancing selectivity, stability, and reusability^28,71^. Competitive adsorption experiments were therefore conducted using equimolar mixed solutions of Cr^6+^, Cu^2^⁺, and Ni^2^⁺ (1000 mg L^−1^ each) at pH 5.8, and the residual equilibrium concentrations (C_e_) are summarized in Table 13. The results clearly demonstrate that metal selectivity is governed by both polymer chemistry and ferrite core composition. Lower C_e_ values indicate stronger adsorption affinity, and across all systems the general selectivity order follows Cr^6+^ > Cu^2^⁺ ≫ Ni^2+^. Cr^6+^ exhibits the lowest residual concentrations (44–108 mg L^−1^), confirming its preferential removal, while Cu^2+^ shows moderate uptake and Ni^2^⁺ consistently displays the weakest adsorption. This trend reflects differences in ionic speciation and surface interactions: at pH ≈ 5.8, Cr^6+^ exists predominantly as anionic chromate species (HCrO_4_^−^/CrO_4_^2^^−^), whereas Cu^2^⁺ and Ni^2^⁺ are hydrated cations. Consequently, electrostatic attraction between anionic Cr^6+^ and positively charged quaternary ammonium groups in LQAA- and CQAA-based MNCs strongly enhances Cr^6+^ adsorption under competitive conditions^72^. LQAA-functionalized MNCs exhibit particularly low Cr^6+^ (C_e_) values and exceptionally low Fe release (down to 0.002 mg L^−1^), indicating efficient adsorption coupled with excellent magnetic core protection. The high segmental mobility and accessibility of LPIL chains facilitate ion exchange while preserving structural integrity, consistent with previous reports^26,73^. CQAA-based systems also maintain high Cr^6+^ and Cu^2^⁺ removal efficiencies, although slightly higher C_e_ values compared to LQAA reflect diffusion limitations imposed by the crosslinked network. Nevertheless, the coexistence of quaternary ammonium and sulfonate groups enables simultaneous binding of anionic and cationic species, supporting effective multimetal adsorption. In contrast, CAA-based hydrogels show higher C_e_ values for all metals, particularly Ni^2^⁺, indicating weaker competitive adsorption. Although carboxylate and sulfonate groups can chelate divalent cations, strong competition from Cr^6+^ and Cu^2^⁺ reduces available binding sites for Ni^2^⁺. Moreover, higher Fe concentrations detected in solution suggest partial destabilization of Fe_3_O_4_ within acidic microenvironments formed inside the hydrogel network, as previously reported for carboxylate-rich magnetic hydrogels^64^. Notably, NiFe_2_O_4_-based MNCs consistently exhibit lower Fe leaching and improved structural stability compared to Fe_3_O_4_ analogues, attributable to the higher chemical robustness of the nickel ferrite spinel structure under mildly acidic and high-ionic-strength conditions. Overall, the extremely low Fe concentrations observed for LQAA- and CQAA-capped MNCs confirm that competitive removal of Cr^6+^, Cu^2^⁺, and Ni^2^⁺ occurs predominantly via adsorption rather than adsorbent degradation, underscoring the environmental safety, stability, and reusability of these systems for multicomponent wastewater treatment.

**Table 13:** Competitive adsorption data of MNCs in the presence 1000 mg/L of each Cr^6+^, Cu^2+^ and Ni^2+^, aqueous solutions at pH 5.8.

MNCs	Adsorption data the heavy metal polluted water						
	Cr^6+^ equilibrium concentrations (C_e_) (mg/L)	q_e Cr_^6+^ (mg.g^-1^)	Cu^2+^ Equilibrium concentrations (C_e_) (mg/L)	q_e Cu_^2+^ (mg g^-1^)	Fe leaching	Ni^2+^ Equilibrium concentrations (C_e_) (mg/L)	q_e Ni_^2+^ (mg g^-1^)
NiFe_2_O_4_.LQAA	107.88	892	622.15	378	0.002	160.58	840
Fe_3_O_4_.LQAA	44.74	955	187.43	812	0.35	205.72	794
NiFe_2_O_4_.CQAA	85.54	914	280.60	719	1.17	108.50	891
Fe_3_O_4_.CQAA	66.015	934	251.96	748	1.28	83.54	916
Fe_3_O_4_.CAA	107.35	893	236.27	763	3.88	315.22	684
NiFe_2_O_4_.CAA	94.99	905	270.03	730	2.24	146.38	853

## Conclusions

Novel MNCs based on Fe_3_O_4_ and NiFe_2_O_4_ nanoparticles capped with PILs, including linear LQAA and crosslinked CQAA networks, were successfully synthesized at room temperature via an in-situ approach. Thermogravimetric analysis indicates that crosslinked polymer networks suppress excessive reduction of metal cations, thereby limiting uncontrolled formation of magnetite and nickel ferrite from single iron species and enhancing thermal stability. TEM and SEM analyses confirm the formation of uniformly dispersed Fe_3_O_4_ and NiFe_2_O_4_ nanoparticles within tailored ionic and hydrogel matrices, demonstrating the effectiveness of quaternized PILs and acrylic acid/AMPS hydrogels as stabilizing and structuring media. In particular, crosslinked systems (Fe_3_O_4_.CQAA and NiFe_2_O_4_.CQAA) exhibit more homogeneous nanoparticle distributions and significantly reduced aggregation compared to linear counterparts. Magnetic measurements reveal that all nanocomposites exhibit typical superparamagnetic behavior with negligible remanence (M_r_) and coercivity (Hc ≈ 0), confirming their soft magnetic nature and suitability for magnetic separation applications. Adsorption kinetics studies show that Cr^6+^ uptake proceeds via a combined mechanism involving film diffusion and intraparticle diffusion rather than a single rate-limiting step. The three-dimensional crosslinked architectures of CQAA and CAA hydrogels facilitate efficient ion transport while maintaining structural integrity, leading to rapid adsorption rates. The overall adsorption performance is governed by the synergistic effects of surface functional groups, polymer architecture, and ferrite core composition. Notably, the developed MNCs exhibit exceptionally high Cr^6+^ adsorption capacities (800–950 mg g^−1^), significantly surpassing most previously reported magnetic adsorbents, underscoring the advantage of PIL-based hydrogel functionalization. Competitive adsorption studies further reveal a clear selectivity trend of Cr^6+^  > Cu^2^⁺ ≫ Ni^2^⁺, driven by strong electrostatic interactions between anionic Cr ions species and quaternized PIL groups. Among the polymer architectures, LQAA-based systems show the highest adsorption efficiency and core stability, followed by CQAA and CAA. Moreover, NiFe_2_O_4_-based nanocomposites consistently outperform Fe_3_O_4_ analogues in terms of chemical stability and resistance to metal leaching, highlighting their suitability for practical wastewater treatment applications.

## Supplementary Information

Supplementary Information.

## Data Availability

The datasets generated and analyzed during the current study are available from the corresponding author upon reasonable request. Code availability Not applicable for that section.
