# Supplementary material for: Removal of toxic hexavalent chromium ions from water using magnetic protic poly (ionic liquids) nanocomposites
Source: Sci Rep. 2026 Jun 11;16:18194. doi: 10.1038/s41598-026-51229-z (PMC13260829; doi:10.1038/s41598-026-51229-z)
Supplement: Supplementary file 1 — Supplementary Information. [file 41598_2026_51229_MOESM1_ESM.docx]

**Removal of Toxic Hexavalent Chromium Cation from Water Using Magnetic Protic Poly (ionic liquids) Nanocomposites**

by

Alia.A.Melegy^a^, S.E. Zaki^a^, S. M. El-Saeed, Nermine E. Maysour^a^,Yasser k. Abdel-Monem^b^, and Ayman M. Atta^a*^

^a^ Petroleum application department, Egyption petroleum research institute, Nasr city, Cairo11727, Egypt

^b^ Menoufia University, Faculty of Science, Chemistry Department, Egypt

^*^Corresponding author: E-mail [aatta@epri.sci.eg](mailto:aatta@epri.sci.eg); [Khaled_00atta@yahoo.com](mailto:Khaled_00atta@yahoo.com)

(a)


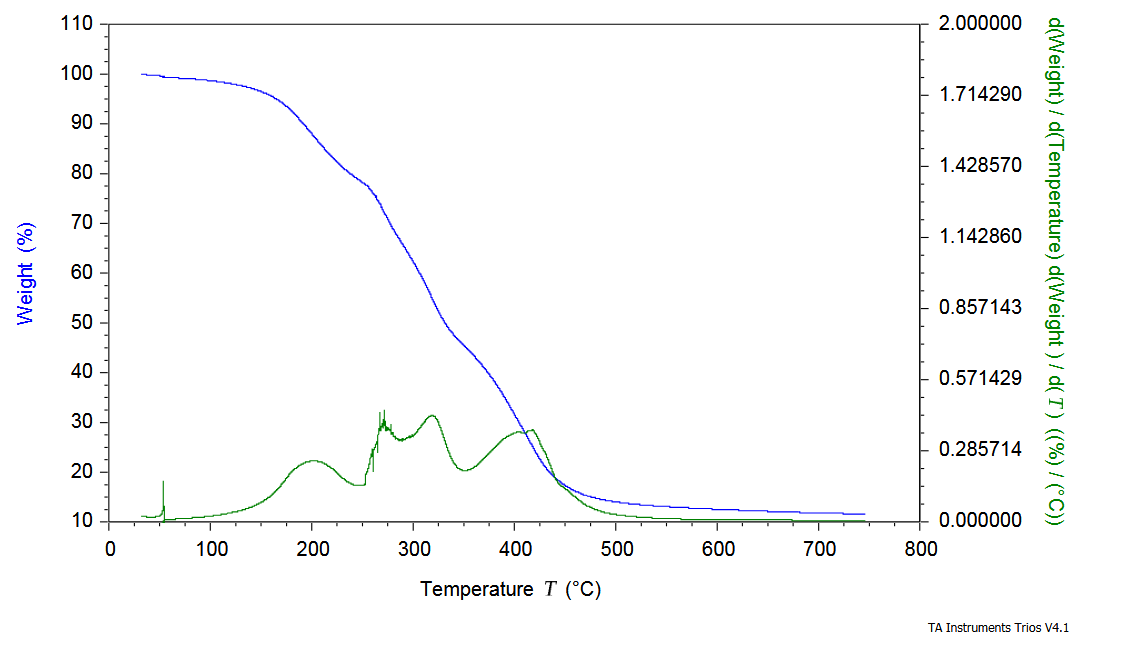


(b)


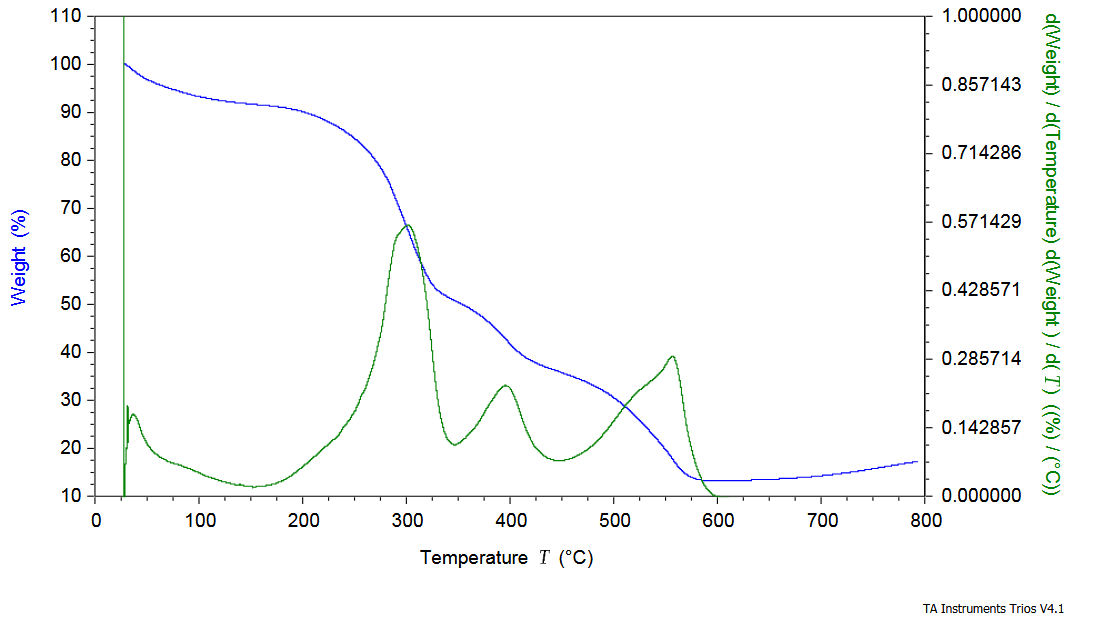


Figure S1. TGA thermograms of a)LQAA and b) CQAA polymers.

(a)

(b)

Figure S2. XRD diffractograms of a) CQAA and b) CAA hydrogels.

Figure S3. Calibration and Uv-visible data of Cr^6+^/ DPC complex after adsorption at optimum conditions using MNCs.

**Figure S4.** Langmuir plot for the adsorption of Cr ^6+^ by a) Fe_3_O_4_.LQAA, b) NiFe_2_O_4_.LQAA, c) Fe_3_O_4_.CQAA, d) NiFe_2_O_4_.CQAA e) Fe_3_O_4_.CAA, f) NiFe_2_O_4_.CAA.

**Figure S5.** Freundlich plot for the adsorption of Cr ^6+^ by a) Fe_3_O_4_.LQAA, b) NiFe_2_O_4_.LQAA, c) Fe_3_O_4_.CQAA, d) NiFe_2_O_4_.CQAA e) Fe_3_O_4_.CAA, f) NiFe_2_O_4_.CAA
